# Supplementary material for: Passive and active immunity in infants born to mothers with SARS-CoV-2 infection during pregnancy: prospective cohort study
Source: BMJ Open. 2021 Jul 7;11(7):e053036. doi: 10.1136/bmjopen-2021-053036 (PMC8264915; doi:10.1136/bmjopen-2021-053036)
Supplement: Supplementary data [file bmjopen-2021-053036supp001.pdf]

PCR Methods:

Nasopharyngeal swab PCR assays.
The PCR test was performed using the following four assays that have been validated and used for clinical diagnostic purpose in our hospital: Xpert® Xpress SARS-CoV-2 assay (Cepheid, Sunnyvale, California, USA), DiaSorin Simplexa™ COVID-19 Direct assay (Diasorin Molecular, Cypress, California, USA), Perkin Elmer® nCoV NAD assay (Perkin Elmer, Waltham, Massachusetts, USA), and Hologic® Aptima™ SARS-CoV-2 Assay (Hologic Inc., Marlborough, Massachusetts, USA).

RNA Extraction**.** Maternal blood, cord blood, placental tissue, and infant meconium RNA was extracted using the QIAmp Viral RNA Mini Kit following the manufacturer's instructions with some adjustments. 300uL of maternal and cord blood in RNAlater (1:1.3 ratio) were used for each extraction. 15-25 mg of placenta and 300 µg of meconium in viral transport media was used for extraction. The kit protocol was followed with buffer amounts scaled up proportionally for the starting amount. RNA was eluted in a 40uL elution buffer for blood and 20uL elution buffer for placenta and meconium. RNA quantity was measured using the Qubit RNA High Sensitivity Assay Kit.

Quantitative real-time PCR**.** Quantitative polymerase chain reaction was performed using the ABI StepOne Plus system. Primer sequences targeted the N (nucleotide) and Orf1b (ORF1b-nsp14) gene. Primer sequences are as follows: forward primer targeting N gene \ (HKU-NF): 5'-TAATCAGACAAGGAACTGATTA-3';Reverse primer (HKU-NR): 5'-CGAAGGTGTGACTTCCATG-3'; and Probe (HKU-NP): 5'-FAM-GCAAATTGTGCAATTTGCGG-TAMRA-3'. Forward primer targeting Orf1b-nsp14 gene (HKU-ORF1b-nsp14F): 5'-TGGGGYTTTACRGGTAACCT-3'; Reverse primer (HKU- ORF1b-nsp14R): 5'-AACRCGCTTAACAAAGCACTC-3'; and Probe (HKU-ORF1b-nsp141P): 5'-FAM-TAGTTGTGATGCWATCATGACTAG-TAMRA-3. RT-qPCR reactions were performed using the TaqMan Fast Virus 1-step Master Mix according to the manufacturer's instructions.

Table 1: PCR Reagents

Reagent	Volume per rxn (µL)
Water (RNAse free)	7.5
TaqMan Fast Virus 1-step (4X)	5
Forward Primer (10 µM)	1
Reverse Primer (10 µM)	1
Prove (10 µM)	0.5
RNA Sample	5

Table 2: PCR Cycle

Steps	Temperature (C)	Time (mm:ss) # cycles
Reverse Transcription	50	05:00
RT Inactivation/denaturation	96	00:20
Amplification	95	00:05:40
Amplification	60	00:30

Table 3: Distribution of severity of maternal symptoms at the time of diagnosis

	**Asymptomatic mothers**	**Mild-moderately symptomatic mothers**	**Severe-critically symptomatic mothers**
	**59**	**78**	**8**
**Time between maternal infection and delivery**			
<60 days, n	50	46	3
60-180 days, n	6	26	5

>180 days, n	3	6	0
**Trimester at the time of maternal infection**			
First Trimester, n	3	8	0
Second Trimester, n	7	12	0
Third Trimester, n	49	58	8
**Trimester at the time of delivery**			
First Trimester, n	0	0	0
Second Trimester, n	0	0	0
Third Trimester, n	59	78	8

Table 4: Delivery specimen PCR results

Participant	Maternal Blood	Cord Blood	Placenta	Infant Meconium
#1, cord IgM 62 RFU	Negative	Negative	Negative	-
#2, cord IgM 65 RFU	-	Negative	Negative	-
#3, cord IgM 136 RFU	-	-	Negative	Negative
