# Supplementary material for: Passive and active immunity in infants born to mothers with SARS-CoV-2 infection during pregnancy: prospective cohort study
Source: BMJ Open. 2021 Jul 7;11(7):e053036. doi: 10.1136/bmjopen-2021-053036 (PMC8264915; doi:10.1136/bmjopen-2021-053036)
Supplement: Author's manuscript [file bmjopen-2021-053036.draft_revisions.pdf]

BMJ Open is committed to open peer review. As part of this commitment we make the peer review history of every article we publish publicly available.

When an article is published we post the peer reviewers' comments and the authors' responses online. We also post the versions of the paper that were used during peer review. These are the versions that the peer review comments apply to.

The versions of the paper that follow are the versions that were submitted during the peer review process. They are not the versions of record or the final published versions. They should not be cited or distributed as the published version of this manuscript.

BMJ Open is an open access journal and the full, final, typeset and author-corrected version of record of the manuscript is available on our site with no access controls, subscription charges or pay-per-view fees (http://bmjopen.bmj.com).

If you have any questions on BMJ Open's open peer review process please email info.bmjopen@bmj.com

BMJ Open

Passive and active immunity in infants born to mothers with SARS-CoV-2 infection during pregnancy: Prospective cohort study

Journal:	*BMJ Open*
Manuscript ID	bmjopen-2021-053036
Article Type:	Original research
Date Submitted by the Author:	01-May-2021
Complete List of Authors:	Song, Dongli; Santa Clara Valley Health and Hospital System, Pediatrics, NICU; Stanford University School of Medicine, Deparment of Pediatrics<br>Prahl, Mary; University of California San Francisco, Department Pediatrics, Division of Pediatric Infectious Diseases and Global Health<br>Gaw, Stephanie; University of California San Francisco, Division of Maternal-Fetal Medicine, Department of Obstetrics, Gynecology and Reproductive Sciences<br>Narasimhan, Sudha Rani; Santa Clara Valley Medical Center, Department of Pediatrics, Division of Neonatology; Stanford University School of Medicine, Deparment of Pediatrics<br>Rai, Daljeet; Stanford University School of Medicine, Department of Family Medicine<br>Huang, Angela; Santa Clara Valley Medical Center, Department of Pediatrics, Division of Neonatology<br>Flores, Claudia; Santa Clara Valley Medical Center, Department of Pediatrics, Division of Neonatology<br>Lin, Christine; University of California San Francisco, Division of Maternal-Fetal Medicine, Department of Obstetrics, Gynecology and Reproductive Sciences<br>Jigmeddagva, Unurzul; University of California San Francisco, Division of Maternal-Fetal Medicine, Department of OBstetrics, Gynceology and Reproduction Sciences<br>Wu, Alan; University of California San Francisco, Department of Laboratory Medicine<br>Warrier, Lakshmi; University of California San Francisco, Department of Medicine<br>Levan, Justine; University of California San Francisco, Department of Medicine<br>Nguyen, Catherine; University of California San Francisco, Department of Medicine<br>Callaway, Perri; University of California San Francisco, Department of Medicine<br>Farrington, Lila ; University of California San Francisco, Department of Medicine<br>Acevedo, Gonzalo; University of California San Francisco, Department of Medicine<br>Gonzalez, Veronica ; University of California San Francisco, Division of Maternal-Fetal Medicine, Department of OBstetrics, Gynecology and Reproductive Sciences

Vaaben , Anna ; University of California San Francisco, Department of Medicine
Nguyen, Phuong; Santa Clara Valley Medical Center, Department of Pathology
Atmosfera , Elda ; Santa Clara Valley Medical Center, Department of Pathology
Marleau, Constance ; Santa Clara Valley Medical Center, Department of Pathology
Anderson, Christina; Santa Clara Valley Medical Center, Department of Pediatrics, Division of Neonatology ; Stanford University School of Medicine, Department of Pediatrics
Misra , Sonya; Santa Clara Valley Medical Center, Department of Pediatrics, Divison of Neonatology ; Stanford University School of Medicine, Department of Pediatrics
Stemmle, Monica ; Santa Clara Valley Medical Center, Department of Pediatrics, Division of Neonatology ; Stanford University School of Medicine, Department of Pediatrics
Cortes, Maria ; Santa Clara Valley Medical Center, Departmetn of Pediatrics, Divsion of Neonatology
McAuley, Jennifer ; Santa Clara Valley Medical Center, Department of Pediatrics, Divison of Neonatology
Metz , Nicole ; Santa Clara Valley Medical Center, Department of Pediatrics, Division of Neonatology
Patel , Rupalee; Santa Clara Valley Medical Center, Department of Pediatrics, Division of Neonatology
Nudelman, Matthew; Marshall University, Department of Pediatrics
Abraham, Susan ; Santa Clara Valley Medical Center, Department of Pediatrics, Division of Neonatology ; Stanford University School of Medicine, Department of Pediatrics
Byrne , James ; Santa Clara Valley Medical Center, Department of Obstetrics and Gynecology, Division of Maternal-Fetal Medicine ; Stanford University School of Medicine, Department of Obstetrics and Gynecology
Jegatheesan, Priya; Santa Clara Valley Health and Hospital System, Pediatrics, Neonatology; Stanford University School of Medicine, Pediatrics

Keywords: COVID-19, IMMUNOLOGY, NEONATOLOGY, Paediatric infectious disease & immunisation < PAEDIATRICS

SCHOLARONE™ Manuscripts

I, the Submitting Author has the right to grant and does grant on behalf of all authors of the Work (as defined in the below author licence), an exclusive licence and/or a non-exclusive licence for contributions from authors who are: i) UK Crown employees; ii) where BMJ has agreed a CC-BY licence shall apply, and/or iii) in accordance with the terms applicable for US Federal Government officers or employees acting as part of their official duties; on a worldwide, perpetual, irrevocable, royalty-free basis to BMJ Publishing Group Ltd ("BMJ") its licensees and where the relevant Journal is co-owned by BMJ to the co-owners of the Journal, to publish the Work in this journal and any other BMJ products and to exploit all rights, as set out in our licence.

The Submitting Author accepts and understands that any supply made under these terms is made by BMJ to the Submitting Author unless you are acting as an employee on behalf of your employer or a postgraduate student of an affiliated institution which is paying any applicable article publishing charge ("APC") for Open Access articles. Where the Submitting Author wishes to make the Work available on an Open Access basis (and intends to pay the relevant APC), the terms of reuse of such Open Access shall be governed by a Creative Commons licence – details of these licences and which Creative Commons licence will apply to this Work are set out in our licence referred to above.

Other than as permitted in any relevant BMJ Author's Self Archiving Policies, I confirm this Work has not been accepted for publication elsewhere, is not being considered for publication elsewhere and does not duplicate material already published. I confirm all authors consent to publication of this Work and authorise the granting of this licence.

For peer review only

Passive and active immunity in infants born to mothers with SARS-CoV-2 infection during pregnancy: Prospective cohort study

Dongli Song, MD[1,2], Mary Prahl, MD[3], Stephanie L. Gaw, MD[4], SudhaRani Narasimhan, MD[1,2], Daljeet Rai, MD[5], Angela Huang, MPH[1], Claudia Flores, BA[1], Christine Y. Lin[4], Unurzul Jigmeddagva, MD[4], Alan H.B. Wu, PhD[6], Lakshmi Warrier[7], Justine Levan, PhD[7], Catherine B.T. Nguyen[7], Perri Callaway[7], Lila Farrington, PhD[7], Gonzalo R. Acevedo, PhD[7], Veronica J. Gonzalez, MD[4], Anna Vaaben, MS[7], Phuong Nguyen, MBA[8], Elda Atmosfera, CLS[8], Constance Marleau, MT[8], Christina Anderson, MD[1,2], Sonya Misra, MD[1,2], Monica Stemmle, MD[1,2], Maria Cortes, DNP[1], Jennifer McAuley, NNP[1], Nicole Metz, BSN[1], Rupalee Patel, DNP[1], Matthew Nudelman[9], Susan Abraham, MD[1,2], James Byrne, MD[10. 11], Priya Jegatheesan, MD[1,2*]

[1]Department of Pediatrics, Division of Neonatology, Santa Clara Valley Medical Center, San Jose, CA, USA

[2]Department of Pediatrics, Stanford University School of Medicine, Stanford, CA, USA

[3]Department of Pediatrics, Division of Pediatric Infectious Diseases and Global Health, University of California, San Francisco, CA, USA

[4]Division of Maternal-Fetal Medicine, Department of Obstetrics, Gynecology and Reproductive Sciences, University of California, San Francisco, CA, USA

[5]Department of Family Medicine, Stanford University School of Medicine, Stanford, CA, USA

[6] Department of Laboratory Medicine, University of California, San Francisco, CA, USA

[7] Department of Medicine, University of California San Francisco, CA, USA

[8]Department of Pathology, Santa Clara Valley Medical Center, San Jose, CA, USA

[9]Department of Pediatrics, Marshall University, Huntington, WV, USA

[10]Department of Obstetrics and Gynecology, Division of Maternal-Fetal Medicine, Santa Clara Valley, CA, USA

[11]Department of Obstetrics and Gynecology, Stanford University School of Medicine, Stanford, CA, USA

For peer review only - http://bmjopen.bmj.com/site/about/guidelines.xhtml

Corresponding Author

Priya Jegatheesan, MD

751 South Bascom Ave, San Jose, CA 95128

Email: Priya.jegatheesan@hhs.sccgov.org

Phone: Mobile 408-687-3493 Office 408-885-5420

For peer review only

Abstract

OBJECTIVE To investigate maternal immunoglobulins' (IgM, IgG) response to SARS-CoV-2 infection during pregnancy and IgG transplacental transfer, to characterize neonatal antibody response to SARS-CoV-2 infection, and to longitudinally follow actively- and passively-acquired SARS-CoV-2 antibodies in infants.

DESIGN A prospective observational study.

SETTING A public healthcare system in Santa Clara County (CA, USA).

PARTICIPANTS Women with SARS-CoV-2 infection during pregnancy and their infants were enrolled between April 15, 2020 and March 31, 2021.

OUTCOMES SARS-CoV-2 serology analyses in the cord and maternal blood at delivery and longitudinally in infant blood between birth and 28 weeks of life.

RESULTS Of 145 mothers who tested positive for SARS-CoV-2 during pregnancy, 86 had symptomatic infections: 78 with mild-moderate symptoms, and eight with severe-critical symptoms. Of the 147 newborns, two infants showed seroconversion at two weeks of age with high levels of IgM and IgG, including one premature infant with confirmed intrapartum infection. The seropositivity rates of the mothers at delivery was 65% (95% CI 0.56-0.73) and the cord blood was 58% (95% CI 0.49-0.66). IgG levels significantly correlated between the maternal and cord blood (R_s= 0.93, p< 0.0001). IgG transplacental transfer ratio was significantly higher when the first maternal positive PCR was 60-180 days before delivery compared to <60 days (1.2 vs. 0.6, p=<0.0001). Infant IgG negative conversion rate over follow-up periods of 1-4, 5-12, and 13-28 weeks were 8% (4/48), 12% (3/25), and 38% (5/13), respectively. The IgG seropositivity in the infants was positively related to IgG levels in the cord blood and persisted up to six months of age.

CONCLUSIONS Maternal SARS-CoV-2 IgG is efficiently transferred across the placenta when infections occur more than two months before delivery. Maternally-derived passive immunity may protect infants up to six months of life. Neonates mount a strong antibody response to perinatal SARS-CoV-2 infection.

For peer review only

Strengths and limitations of this study

- This study included pregnant mothers with SARS-CoV-2 infection in all three trimesters of pregnancy and provided a comprehensive understanding of maternal SARS-CoV-2 IgG transplacental transfer throughout pregnancy.
- This is the first longitudinal study that has followed maternally-derived SARS-CoV-2 IgG in infants up to 28 weeks.
- This is the first study, to our knowledge, that characterized neonatal serology response to perinatal SARS-CoV-2.
- In asymptomatic mothers the first positive PCR might not represent the precise timing of infection.
- The cohort had few severe cases of maternal infection and premature births before 35 weeks of gestation.

Funding statement

This works was supported by Bill and Melinda Gates Foundation (INV-017035 to SLG), National Institutes of Health (NIAID K08AI141728 to SLG, NIAID K23AI127886 to MP), Marino Family Foundation, and Valley Medical Center Foundation. The funders did not have any role in the study design, patient recruitment, data collection, analysis, or interpretation of the results. All the authors had full access to the full data in the study and accept responsibility to submit for publication.

Competing interests' statement

None declared.

Author contributions

For peer review only

D.S. conceptualized and designed the project, participated in patient enrollment, sample collection, data visualization and interpretation, and wrote the manuscript draft.

P.J. conceptualized and designed the project, participated in patient enrollment, sample collection, data collection, analysis, interpretation, and edited manuscript.

M.P., S.L.G designed collection and processing protocols, performed sample processing, oversaw experiments and data analysis, provided funding, edited the manuscript.

S.R.N. conceptualized and designed the project, participated in patient enrollment, sample collection, data collection and edited manuscript.

D.R. participated in study design, patient enrollment, and sample collection.

A.H. coordinated data collection and management, participated in sample collection, and oversaw the implementation of the project.

C.V.F. participated in patient enrollment, coordinated sample collections, processing, and data management

L.W. designed collection and processing protocols, performed sample processing and data collection.

J.L. and C.B.T.N., CYL, UJ, VJG designed and performed experiments, performed sample processing and data collection.

P.C., L.F, G.R.A., A.V. optimized collection and processing protocols, performed sample processing and data collection

A.H.B.W. designed and oversaw serology assays.

E.A., P.N., C.M. oversaw sample collections and processing.

C.A., S.M., M.S., M.C., J.M., S.A., N.M. participated in patient enrollment and sample collection

M.N. participated in data analysis and preparing the figures for the manuscript.

R.P. participated in the study implementation, and sample collection.

J.B. designed, and oversaw the patient recruitment and implementation of the project

All authors reviewed and approved the manuscript.

For peer review only

Data sharing statement:

De-identified data is published in Mendeley data sharing site and available in the following doi:10.17632/6scfwt55fd.2.

Word count

2995 words

For peer review only

Introduction

An important aspect of immunity against infectious pathogens in young infants relies on effective maternal antibody production, transfer of maternal antibodies across the placenta to the fetus, and persistence of passive immunity in the infant. Our understanding of the immune response to severe acute respiratory syndrome coronavirus-2 (SARS-CoV-2) is expanding rapidly through extensive basic and clinical studies.[1-4] However, the literature on SARS-CoV-2 immunity in pregnant mothers and infants remains limited.[5-9] Global efforts are focused on controlling the COVID-19 pandemic through public health prevention measures and universal vaccination. Knowledge of neonatal immune response to SARS-CoV-2 and maternally-derived passive immunity in young infants is urgently needed to guide ongoing COVID-19 infection prevention and vaccination strategies in pregnant mothers and infants.

Recent publications have shown evidence of maternal SARS-CoV-2 antibody transplacental transfer.[6,7,9] However, the majority of maternal SARS-CoV-2 infections in these reports occurred late in pregnancy, as these studies were conducted during the first few months of the COVID-19 pandemic. Therefore, the timing and efficiency of maternal antibody production and transplacental transfer throughout gestation remain to be fully understood, which has important implications for the timing of maternal immunization to benefit both pregnant mothers and their young infants. Furthermore, the important question as to the persistence of maternally-derived passive immunity in infants needs to be investigated. While SARS-CoV-2 infection has been described in newborns,[10,11] little is known about infant immune response to perinatal infection. The aims of this study were to investigate SARS-CoV-2 antibody transplacental transfer with respect to the timing of maternal infection during gestation, antibody response to SARS-CoV-2 infection in the newborns, and persistence of passively- and actively-acquired SARS-CoV-2 antibodies in infants.

Methods:

Study design, participants, and procedures

For peer review only

This is a prospective observational study of pregnant mothers with SARS-CoV-2 infection during pregnancy and their infants. The study was conducted from April 15, 2020 to March 31, 2021, in a public healthcare system, including one regional medical center and two community hospitals. The healthcare system primarily serves the medically indigent population of Santa Clara County California (USA). The study was approved by the institutional review boards of Santa Clara Valley Medical Center. Patients or the public were not involved in the design, or conduct, or reporting, or dissemination plans of our research.

In April 2020, our health system implemented a universal screening protocol for SARS-CoV-2 infection in women presenting in labor or within three days prior to admission for elective deliveries.[12] SARS-CoV-2 infection was diagnosed based on a positive SARS-CoV-2 reverse transcriptase polymerase chain reaction (RT-PCR) test using nasopharyngeal specimen performed either before delivery or through universal screening at delivery. The timing of maternal SARS-CoV-2 infection was based on the first positive SARS-CoV-2 PCR test. The severity of SARS-CoV-2 symptoms (mild, moderate, severe, or critical) was assessed according to the Society for Maternal-Fetal Medicine guidelines.[13] If the maternal infection was within 10-14 days of delivery, the mother and infant roomed in together with airborne isolation precautions and the mother wore a surgical mask when holding and breastfeeding the baby during the isolation period. The nasopharyngeal SARS-CoV-2 PCR was performed in the newborns at 24 hours of life. The infants were retested between 48-72 hours of life if they were in the Neonatal Intensive Care Unit (NICU).

Maternal and cord blood were collected at the time of delivery. Serial infant blood samples were collected between the ages of 1-28 weeks, coordinated with their clinic visits. Levels of SARS-CoV-2 immunoglobulin M (IgM) and immunoglobulin G (IgG) to the spike protein receptor binding domain (RBD) and nucleocapsid protein (NP) of SARS-CoV-2 were measured using the Pylon 3D automated immunoassay system (ET Healthcare, Palo Alto, CA) as previously described.[14] The background

For peer review only

corrected signal was reported as relative fluorescent units (RFU), which is proportional to the amount of specific antibodies in the sample allowing for quantification. The positive cutoffs for IgM and IgG were set to >50 RFU to achieve 100% specificity and a high level of sensitivity.[14] Quantitative reverse transcriptase PCR (qRT-PCR) was performed on maternal blood, cord blood, placenta, and meconium in a subset of infants. Primer sequences targeted the N and Orf1b SARS-CoV-2 genes (supplemental Methods, supplemental Tables 1 and 2).

Data collection and analysis

Clinical data included maternal and neonatal demographics, the severity of maternal symptoms of SARS-CoV-2 infection, days between maternal first positive SARS-CoV-2 PCR test and delivery, and neonatal outcomes. Demographics, clinical outcomes, and serum IgM and IgG levels were summarized using descriptive analyses. Transplacental IgG transfer ratios were calculated by dividing cord blood IgG levels by maternal blood IgG levels. Correlation between maternal and cord blood IgG levels and correlation between placental transfer ratio and gestational age (GA) at birth were analyzed using Spearman's rank-order correlation. The transfer ratios were compared between maternal groups based on infection severity and time between first maternal positive PCR and delivery using the Kruskal-Wallis test, followed by Dunn's test for pairwise multiple comparisons with the Holm-Sidák stepwise adjustment.

Results

During the study period, 3936 mothers delivered in the health system with 3956 live births, and 254 (6.5%) of the mothers had at least one positive SARS-CoV-2 PCR test during the pregnancy. The study enrolled 145 mothers with SARS-CoV-2 infection and 147 of their infants (Figure 1). Of 145 enrolled mothers, 86 (59%) had symptomatic infection, including 78 with mild-moderate symptoms and eight with severe-critical symptoms (Table 1). The distribution of the severity of the maternal infection is shown in supplemental Table 3. Of 147 newborns, 23 (16%) were admitted to the NICU. SARS-CoV-2 PCR was

For peer review only

performed on nasopharyngeal specimens of 89 newborns at 24 hours of life, and only one 31-week preterm infant tested positive.

Maternal and cord blood serology

Serum serology was performed on 129 mothers at delivery and 144 cord blood samples. The temporal profiles of maternal and cord blood IgM and IgG with respect to the timing of first maternal PCR positivity are shown in Figure 2. Antibody status and levels in maternal and cord blood were evaluated in four groups based on the days between maternal first positive SARS-CoV-2 PCR and delivery (<14 days, 14 to 59 days, 60 to 180 days, and >180 days) (Table 2). The maternal seropositivity rate at delivery was 65% (84/129, 95% CI 0.56-0.73), and the cord blood IgG positivity rate was 58% (83/144, 95% CI 0.49-0.66).

Paired serology analysis was performed in 125 maternal-cord blood samples (Table 2). Of the 77 IgG positive mothers, 69 (90%) of their newborns' cord sera were positive for IgG. Of the eight IgG negative infants, seven were born to mothers with infection within 45 days of delivery, and one was born to a mother who had a positive PCR at 254 days before delivery. Of the 48 IgG negative mothers, 45 (94%) of their newborns' cord sera were negative for IgG. Notably, three infants whose cord blood was positive for IgM (65, 136, and 62 RFU) were born to mothers whose blood was also positive for IgM at the time of delivery. The follow-up serology tests for two of the infants at two and three weeks of age were negative for IgM and IgG. No follow-up serology was available for the third infant. Available delivery specimens (maternal and cord blood, placenta, and meconium) were evaluated by SARS-CoV-2 PCR and found to be negative for all three infants (supplemental Table 4).

There was a significant positive correlation between IgG levels in the 125 paired maternal and cord blood samples ($R_s=0.93$, $p<0{\cdot}0001$, Figure 3A). Transplacental IgG transfer ratios were calculated in 77 IgG

For peer review only

positive mother-infant dyads, and the median transfer ratio was 1.0 (95% CI 0.86-1.09). The transfer ratio was significantly higher in the mothers who were severe-critically symptomatic (n=4) compared to mothers who were asymptomatic (n=23) (1.6 vs. 1.0, p=0.003) or mild-moderately symptomatic (n=50) (1.6 vs. 0.9, p=0.002). To illustrate the temporal effect of maternal infection on transfer efficiency, we analyzed transfer ratios of 54 symptomatic mother-infant dyads. Asymptomatic mothers were excluded from this analysis as their timing of infections cannot be concluded definitively from the timing of PCR positivity (Figure 3B). The transfer ratios based on time elapsed from the first maternal positive PCR to delivery were 0.6 (<60 days, n=22), 1.2 (60-180 days, n=27), and 0.9 (>180 days, n=5). The ratio was significantly higher in the 60-180 days group compared to the <60 days group (1.2 vs. 0.6, p=<0.0001). Transfer ratios based on the trimester of maternal infection were 0.9 (1st trimester, n=7), 1.2 (2nd trimester, n=9), and 0.9 (3rd trimester, n=38) (Figure 3D). The ratio was significantly higher in second trimester infections than third trimester infections (1.2 vs. 0.9, p=0.02). There was no significant correlation between the transfer ratio and GA at birth (Rs=0.18, p=0.1, Figure 3C); however, 95% of the infants in our cohort were born at greater than 34 weeks gestation.

Maternally-derived IgG longitudinal follow-up in infants

To evaluate maternally-derived IgG persistence postnatally, we followed serology in 48 infants with positive cord IgG. All infants showed a steady decrease in IgG levels over time (Figure 4A). The IgG seroconversion rate was calculated for those infants who had at least one serology test during the follow-up age periods of 1-4 weeks, 5-12 weeks, and 13-28 weeks. The negative IgG conversion rates for the three follow-up periods were 8% (4/48), 12% (3/25), and 38% (5/13), respectively. The infants who had lower levels of IgG in the cord blood became IgG negative earlier; the cord IgG levels of those infants who seroconverted during the three follow-up periods ranged between 52-66 RFU, 68-150 RFU, and 123-251 RFU, respectively. Two infants who had cord IgG levels greater than 500 RFU remained seropositive at 27 weeks of age.

For peer review only

Infant antibody response to perinatal SARS-CoV-2 infection

We performed surveillance serology tests at 2-4 weeks of age in 23 of 41 (56%) infants who had negative serology in the cord blood and were born to mothers with first positive PCR <14 days before delivery. Two infants showed seroconversion, including the 31-week preterm infant who tested positive for SARS-CoV-2 and a term infant. Interestingly, both infants were born to asymptomatic mothers who tested positive for SARS-CoV-2 PCR for the first time at delivery and negative for SARS-CoV-2 antibodies, indicating a new onset of infection. Both infants were asymptomatic for SARS-CoV-2 infection. The preterm infant, born to a mother with spontaneous preterm labor, was admitted to the NICU immediately after birth, isolated from the mother for 14 days, and discharged home at 17 days of life after an unremarkable NICU course. The infant's cord blood SARS-VoC-2 PCR was negative, but nasopharyngeal PCR was positive at 24 hours of life and remained positive at discharge. Additionally, the infant's meconium and maternal blood at the time of delivery were PCR positive. The term infant roomed in with the mother in the postpartum unit and was discharged home at two days of life. This infants' cord blood and nasopharyngeal SARS-CoV-2 PCR were negative at 24 hours of life, and nasopharyngeal PCR was not repeated.

The preterm infant showed serial negative serology tests after birth on days two, four, and eight, then seroconverted on day 16 (IgM 1548 RFU, IgG 335 RFU) (Figure 4B). The infant's IgM decreased to 134 RFU, and IgG increased to 1873 RFU at eight weeks. The term infant had the first follow-up test at two weeks and was found positive for IgM (225 RFU) and IgG (80RFU) (Figure 4C). The infant's IgM became negative, and IgG peaked at 1841 RFU at eight weeks; the IgG subsequently decreased to 648 RFU at 24 weeks.

Discussion

We conducted a prospective observational study in 145 pregnant mothers with SARS-CoV-2 infections during pregnancy and 147 of their infants. The majority of infected mothers seroconverted before

For peer review only

delivery. The IgG levels in maternal blood at delivery and cord blood were highly correlated. High transplacental IgG transfer ratios were observed when infection onset was greater than 60 days prior to delivery or in the second trimester. The persistence of maternal-derived IgG in infants was positively correlated to the initial cord blood level. Additionally, we showed strong antibody responses to perinatal SARS-CoV-2 infection in two asymptomatic neonates.

In our study, 6.5% of mothers presenting for delivery had at least one positive SARS-CoV-2 PCR during their current pregnancy. The majority of mothers had asymptomatic or mild-moderate infections, consistent with previous cohort studies.[15,16] The maternal IgG levels at delivery were relatively low, comparable to levels in non-ICU patients.[14] Importantly, the temporal profiles of maternal and cord blood IgG levels were in parallel, peaking around 60-90 days post maternal infection. The timing and efficiency of maternal IgG transfer have important implications for developing maternal immunization strategies to protect infants.[17-19] For example, in maternal pertussis immunization, infant seropositivity rate and cord blood IgG levels to pertussis toxin were higher following Tdap immunization during the second trimester than during the third trimester. We studied pregnant mothers who had SARS-CoV-2 infections in all three trimesters and provide a comprehensive profile of transplacental IgG transfer with respect to the timing of infections throughout gestation. We observed that transfer ratio was 0.6 when infection onset was less than 60 days before delivery; plateaus at 1.2 and 0.9 when infections occurred 60-180 days and greater than 180 days before delivery. Prior studies of pregnancy related infection in the last 70 days of gestation found impaired SARS-CoV-2 IgG transplacental transfer (ratio 0.7).[7,8] Another study characterized a cohort of pregnant mothers who had infections during the last 120 days of gestation and showed that transfer ratios increased with length of time from infection to delivery, with transfer ratios reached above 1.0 in the majority of mothers.[6] Taken together, these studies demonstrate that cross-placental SARS-CoV-2 IgG transfer occurs throughout gestation, and a higher transfer efficiency is achieved when infection onset is more than two months prior to delivery. Matching the peak IgG transplacental transfer

For peer review only

and the peak immune response after maternal infection may result in high cord IgG. Information from these maternal and cord serology studies is important for guiding the timing of maternal vaccination in pregnancy to optimize neonatal immunity in concert.

In our study, the persistence of maternal-derived IgG in infants showed a wide range, from two weeks to more than 26 weeks of age. An important observation is that IgG positivity in infants is positively associated with the initial cord IgG levels that are determined by maternal IgG levels and transplacental transfer efficiency. As more pregnant mothers are vaccinated for SARS-CoV-2, knowledge of passive immunity in infants may inform mother-infant care and SARS-CoV-2 vaccination strategy in infants.

Consistent with prior literature showing rare vertical maternal-fetal transmission,[20-23] we found only one infant with confirmed intrapartum acquired neonatal infection. (21) This infant was seronegative in cord blood and during the first week of life but seroconverted at two weeks of life, providing insight into the timing of infant seroconversion in the setting of intrapartum infection. We identified another infant who seroconverted at two weeks follow-up test; however, available virology and serology data is not sufficient to determine the timing and mode of this perinatal infection. Clinical presentations of perinatal SARS-CoV-2 infection have been described previously;[10,11 24] however, little is known about neonatal serology response and long-term clinical outcomes. Interestingly, both infants in our study had asymptomatic infection but mounted strong antibody responses; the timing of seroconversion and levels of IgM and IgG are comparable to that observed in adult patients with severe disease.[14] Both infants remained asymptomatic in the first months of life. Their long-term clinical outcomes, along with immune status, will be followed. Additionally, these two cases highlight the increased risk for perinatal SARS-CoV-2 infection in infants born to mothers who have new-onset infections around the time of delivery,[10] with implications for developing targeted protection measures and postnatal antibody screening for high-risk newborns. In our study, three infants were positive for IgM in cord blood but negative for SARS-CoV-2 virologically in birth specimens and negative for IgM and IgG at two and three weeks of age, suggesting

For peer review only

these transient IgM levels may be false positives or maternal blood contamination. There were two prior case reports describing similar transient positive IgM levels in the cord blood without virological evidence of infection.[25,26] Thus, diagnosis of congenital SARS-CoV-2 infection cannot be made based solely on the presence of IgM in the cord blood.[27-30]

This maternal-infant serology study, one of the largest cohorts to date, included pregnant mothers with SARS-CoV-2 infection in all three trimesters of pregnancy and provided a more comprehensive understanding of maternal SARS-CoV-2 IgG transplacental transfer. This is the first longitudinal study that has followed the level of maternally-derived SARS-CoV-2 IgG in infants up to 28 weeks and neonatal serology response after perinatal SARS-CoV-2 infection up to 24 weeks. Another strength of the study is that the cohort is representative of COVID-19 in the community. Over 90% of the mothers in this cohort are Hispanic, a population highly impacted by the COVID-19 pandemic. Our study has several limitations. It was conducted in a single healthcare system. The timing of infection was based on the first positive PCR, which might not represent the precise timing of infection in asymptomatic mothers. Our cohort had few severe cases and premature births before 35 weeks of gestation. Universal screening at the time of admission also introduces a bias in the identification of asymptomatic SARS-CoV-2 cases at or near-term gestation, as the universal screening was not implemented in our prenatal care visits and asymptomatic screening was not readily available in our general community during the study period.

For peer review only

Conclusion

Our study provides insights into the intricate connections between the timing of maternal SARS-CoV-2 infection, dynamics of maternal antibody production, and transplacental immunity transfer. These processes determine the level of maternally-derived IgG in infants at birth, which in turn affects persistence of passive immunity in infants. Neonates are capable of mounting strong serology responses to perinatal SARS-CoV-2 infection. These findings have important implications in determining optimal

timing of vaccination in pregnant mothers and infants. Future investigations are needed to address the durability and protection of passively and actively acquired antibodies in the infant.

Acknowledgements

Thank you to Dr. Margaret Feeney for support of these experiments and Robin D. Wu who reviewed the manuscript and provided helpful comments and suggestions. We thank the mothers, newborns and their families who participated in the study; staff and providers in Labor and Delivery, Post-partum unit, NICU, pathology and laboratory services, at Santa Clara Valley Medical Center and O'Connor Hospital; outpatient pediatrics clinic, and BRIDGE home follow up program. We thank First 5 of Santa Clara County for their support.

References

1. Blanco-Melo D, Nilsson-Payant BE, Liu WC, et al. Imbalanced Host Response to SARS-CoV-2 Drives Development of COVID-19. *Cell.* 2020;181(5):1036-1045.e1039.
2. Lucas C, Wong P, Klein J, et al. Longitudinal analyses reveal immunological misfiring in severe COVID-19. *Nature.* 2020;584(7821):463-469.
3. Briere CE, Lucas R, McGrath JM, Lussier M, Brownell E. Establishing breastfeeding with the late preterm infant in the NICU. *J Obstet Gynecol Neonatal Nurs.* 2015;44(1):102-113.
4. Stephens DS, McElrath MJ. COVID-19 and the Path to Immunity. *JAMA.* 2020;324(13):1279-1281.
5. Flannery DD, Gouma S, Dhudasia MB, et al. SARS-CoV-2 seroprevalence among parturient women in Philadelphia. *Sci Immunol.* 2020;5(49).
6. Flannery DD, Gouma S, Dhudasia MB, et al. Assessment of Maternal and Neonatal Cord Blood SARS-CoV-2 Antibodies and Placental Transfer Ratios. *JAMA Pediatr.* 2021.
7. Edlow AG, Li JZ, Collier AY, et al. Assessment of Maternal and Neonatal SARS-CoV-2 Viral Load, Transplacental Antibody Transfer, and Placental Pathology in Pregnancies During the COVID-19 Pandemic. *JAMA Netw Open.* 2020;3(12):e2030455.
8. Atyeo C, Pullen KM, Bordt EA, et al. Compromised SARS-CoV-2-specific placental antibody transfer. *Cell.* 2021;184(3):628-642.e610.
9. Kubiak JM, Murphy EA, Yee J, et al. Severe acute respiratory syndrome coronavirus 2 serology levels in pregnant women and their neonates. *Am J Obstet Gynecol.* Epub January 23, 2021.

For peer review only

10. Woodworth KR, Olsen EO, Neelam V, et al. Birth and Infant Outcomes Following Laboratory-Confirmed SARS-CoV-2 Infection in Pregnancy - SET-NET, 16 Jurisdictions, March 29-October 14, 2020. *MMWR Morb Mortal Wkly Rep.* 2020;69(44):1635-1640.

11. Raschetti R, Vivanti AJ, Vauloup-Fellous C, Loi B, Benachi A, De Luca D. Synthesis and systematic review of reported neonatal SARS-CoV-2 infections. *Nat Commun.* 2020;11(1):5164.

12. Yang S, Bhatnagar R, Byrne J, Jelks A. Universal testing for severe acute respiratory syndrome coronavirus 2 upon admission to three labor and delivery units in Santa Clara County, CA. *Clinical Journal of Obstetrics and Gynecology.* 2020;3:109-113.

13. Halscott TM, MS, Vaught JM, Miller EM, MPH. Management Considerations for Pregnant Patients with Covid-19. 2020;October 9. https://www.smfm.org/covidclinical.

14. Lynch KL, Whitman JD, Lacanienta NP, et al. Magnitude and Kinetics of Anti-Severe Acute Respiratory Syndrome Coronavirus 2 Antibody Responses and Their Relationship to Disease Severity. *Clin Infect Dis.* 2021;72(2):301-308.

15. Sutton D, Fuchs K, D'Alton M, Goffman D. Universal Screening for SARS-CoV-2 in Women Admitted for Delivery. *N Engl J Med.* 2020;382(22):2163-2164.

16. Adhikari EH, Moreno W, Zofkie AC, et al. Pregnancy Outcomes Among Women With and Without Severe Acute Respiratory Syndrome Coronavirus 2 Infection. *JAMA Netw Open.* 2020;3(11):e2029256.

17. Fouda GG, Martinez DR, Swamy GK, Permar SR. The Impact of IgG transplacental transfer on early life immunity. *Immunohorizons.* 2018;2(1):14-25.

18. Abu Raya B, Edwards KM, Scheifele DW, Halperin SA. Pertussis and influenza immunisation during pregnancy: a landscape review. *Lancet Infect Dis.* 2017;17(7):e209-e222.

19. Eberhardt CS, Blanchard-Rohner G, Lemaître B, et al. Maternal Immunization Earlier in Pregnancy Maximizes Antibody Transfer and Expected Infant Seropositivity Against Pertussis. *Clin Infect Dis.* 2016;62(7):829-836.

20. Salvatore CM, Han JY, Acker KP, et al. Neonatal management and outcomes during the COVID-19 pandemic: an observation cohort study. *Lancet Child Adolesc Health.* 2020;4(10):721-727.

21. Gale C, Quigley MA, Placzek A, et al. Characteristics and outcomes of neonatal SARS-CoV-2 infection in the UK: a prospective national cohort study using active surveillance. *Lancet Child Adolesc Health.* 2021;5(2):113-121.

22. Flaherman VJ, Afshar Y, Boscardin J, et al. Infant Outcomes Following Maternal Infection with SARS-CoV-2: First Report from the PRIORITY Study. *Clin Infect Dis.* 2020.

23. Dumitriu D, Emeruwa UN, Hanft E, et al. Outcomes of Neonates Born to Mothers With Severe Acute Respiratory Syndrome Coronavirus 2 Infection at a Large Medical Center in New York City. *JAMA Pediatr.* 2021;175(2):157-167.

24. Wu YT, Liu J, Xu JJ, et al. Neonatal outcome in 29 pregnant women with COVID-19: A retrospective study in Wuhan, China. *PLoS Med.* 2020;17(7):e1003195.

For peer review only

25. Zeng H, Xu C, Fan J, et al. Antibodies in Infants Born to Mothers With COVID-19 Pneumonia. *JAMA.* 2020.

26. Dong L, Tian J, He S, et al. Possible Vertical Transmission of SARS-CoV-2 From an Infected Mother to Her Newborn. *JAMA.* 2020.

27. Shah PS, Diambomba Y, Acharya G, Morris SK, Bitnun A. Classification system and case definition for SARS-CoV-2 infection in pregnant women, fetuses, and neonates. *Acta Obstet Gynecol Scand.* 2020;99(5):565-568.

28. Lamouroux A, Attie-Bitach T, Martinovic J, Leruez-Ville M, Ville Y. Evidence for and against vertical transmission for severe acute respiratory syndrome coronavirus 2. *Am J Obstet Gynecol.* 2020;223(1):91.e91-91.e94.

29. Fenizia C, Biasin M, Cetin I, et al. Analysis of SARS-CoV-2 vertical transmission during pregnancy. *Nat Commun.* 2020;11(1):5128.

30. Kimberlin DW, Stagno S. Can SARS-CoV-2 Infection Be Acquired In Utero?: More Definitive Evidence Is Needed. *JAMA.* 2020.

For peer review only

Figure 1: Study participants enrollment

Figure 2: Temporal distribution of maternal and cord blood IgM and IgG

Panel A, B. scatterplots show the distribution of maternal blood SARS-CoV-2 IgM and IgG levels in relative fluorescent unit (RFU) at the time of delivery in Y-axis and days from maternal first positive SARS-CoV-2 PCR test to delivery in X-axis. Panel C, D scatterplots show the distribution of cord blood SARS-CoV-2 IgM and IgG levels in RFU at the time of delivery in Y-axis and days from maternal first positive SARS-CoV-2 PCR test to delivery in X-axis. The different colors represent the severity of the maternal symptoms at the time of diagnosis.

Figure 3: Correlation of cord blood and maternal IgG and distribution of IgG transplacental transfer ratio

Panel A. Scatterplot shows the correlation of cord blood SARS-CoV-2 IgG levels in Y-axis and maternal blood SARS-CoV-2 IgG levels in X-axis in relative fluorescent unit (RFU). Panel B. Scatterplot shows the distribution of IgG transplacental ratio (cord blood/maternal blood SARS-CoV-2 IgG levels) in the Y-axis and days from maternal first positive SARS-CoV-2 PCR test to delivery in X-axis. Panel C. Scatterplot shows the distribution of IgG transplacental ratio in the Y-axis and gestational age at the time of delivery in X-axis. Panel D. Scatterplot shows the distribution of IgG transplacental ratio in the Y-axis and gestational age at the time of maternal first positive SARS-CoV-2 PCR test in X-axis. The different colors represent the severity of the maternal symptoms at the time of diagnosis.

Figure 4: Longitudinal follow-up of SARS-CoV-2 antibody levels in infants

Panel A shows the longitudinal IgG levels of the infants who had cord blood IgG level >50 relative fluorescent unit (RFU). The infants' IgG levels in RFU is shown in Y-axis, and the age of the infant in weeks at the time of follow-up is shown in X-axis. The infants whose IgG became negative, <50RFU, during the longitudinal follow up are shown in red color. Panel B shows the IgG and IgM levels of the term infant whose cord antibody was negative and seroconverted at 2 weeks of life. Panel C shows the

For peer review only

IgG and IgM levels of the 31 weeks preterm infant with confirmed intrapartum SARS-CoV-2 infection whose cord antibody was negative and seroconverted at 2 weeks of life.

For peer review only

Table 1. Maternal and neonatal demographics and outcomes

	**Maternal and infant serology cohort**
Mothers, n	145
Newborns, n	147
**Maternal demographics and outcomes**	
Maternal age, years, median (range)	27 (16, 42)
Gravida, median (range)	3 (1, 12)
Para, median (range)	1 (0, 9)
Hispanic, n (%)	126 (87)
Race, n (%)	
White, n (%)	130 (90)
Black, n (%)	6 (4)
Asian, n (%)	9 (6)
Asymptomatic, n (%)	59 (41)
Mild to moderately symptomatic, n (%)	78 (54)
Severe to critically symptomatic, n (%)	8 (6)
Symptomatic at the time of diagnosis, n (%)	86 (59)
Symptomatic at the time of delivery, n (%)	22 (15)
Cesarean section, n (%)	46 (32)
Multiple pregnancies, n (%)	3 (2)
Maternal diabetes, n (%)	29 (20)
Maternal hypertension, n (%)	30 (21)
Maternal obesity, n (%)	33 (23)
Preterm delivery, n (%)	15 (10)

For peer review only

Intrauterine fetal demise, n (%)	1 (1)
**Neonatal demographics and outcomes**	
Gestational age, weeks, median (range)	39.1 (27.4, 41.6)
Birth weight, grams, median (range)	3285 (990, 4670)
Breastfeeding in the hospital, n (%)	143 (97)
Exclusive breastfeeding in the hospital, n (%)	85 (58)
Rooming in with mother, n (%)	132 (90)
NICU admission, n (%) [a]	23 (16)
Length of stay during birth hospital, days, median (range)	2 (1, 81)
SARS-CoV-2 nasopharyngeal swab positive, n (%) [b]	1 (1)

[a]Reasons for NICU admissions: 7 for prematurity, 1 for a congenital anomaly, 1 for dehydration and 14 for respiratory distress, metabolic acidosis and or evaluation for infection.
[b]SARS-COV-2 PCR using nasopharyngeal specimens was performed in 70 (99%) of the newborns born to mothers who were first PCR positive within two weeks of delivery.

For peer review only

Table 2. Maternal and cord blood serology and timing of maternal first positive PCR

	Total	2w<br>0-13d	2w-2m<br>14-59d	2m-6m<br>60-179d	>6m<br>≥180d
**Maternal serology, n**	**129**	**56**	**28**	**36**	**9**
IgM- and IgG-, n	45	35	4	5	1
IgM+ and IgG-, n	4	4	0	0	0
IgM+ and IgG+, n	29	6	13	9[a]	1[b]
IgM- and IgG+, n	51	11	11	22	7
IgM+ and/or IgG+, n	84	21	24	31	8
IgM, RFU, median (range)	27 (7, 1388)	25·5 (2, 315)	34.5 (7, 1388)	26.5 (11, 263)	25 (7, 59)
IgG, RFU, median (range)	84 (1, 3582)	22·5 (1, 401)	178 (1, 1123)	194.5 (22, 2311)	199 (41, 3582)
**Cord blood serology, n**	**144**	**70**	**27**	**38**	**9**
IgG-, n	61	48	8	4	1
IgG+, n	83	22	18	32	8
IgG, RFU, median (range)	66.5 (0, 2916)	14 (0, 1820)	77 (2, 1164)	232 (22, 2916)	209 (45, 1173)
**Paired cord and maternal blood serology, n**	**125**	**54**	**26**	**36**	**9**
Maternal IgG + and Cord blood IgG +, n	69	12	19	31	7
Maternal IgG + and cord blood IgG -, n	8	4	3	0	1
Maternal IgG – and cord blood IgG -, n	45	37	4	4	0
Maternal IgG – and cord blood IgG, +, n	3	1	0	1	1

RFU=Relative fluorescent unit.
[a] All 9 mothers' first positive SARS-VoC-2 PCR were between 63 and 103 days before delivery.
[b] This mother's SARS-VoC-2 PCR was positive at 10 weeks gestation and was positive again at the time of delivery at 39 weeks gestation.

For peer review only

For peer review only

Figure 1

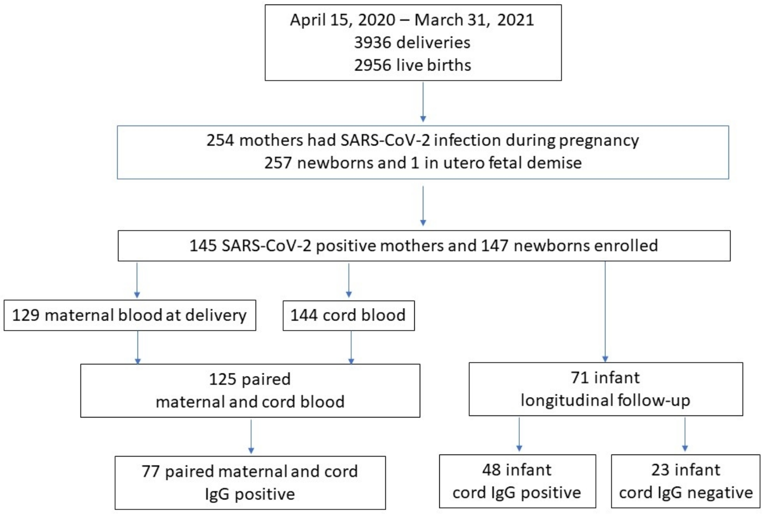

Figure 1. Study participants enrollment

338x190mm (96 x 96 DPI)

Figure 2

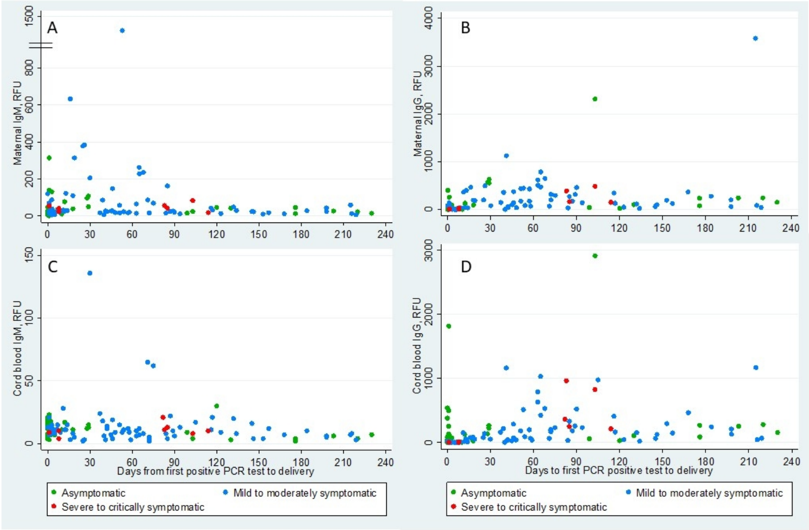

Figure 2. Temporal distribution of maternal and cord blood IgM and IgG
Panel A, B. scatterplots show the distribution of maternal blood SARS-CoV-2 IgM and IgG levels in relative fluorescent unit (RFU) at the time of delivery in Y-axis and days from maternal first positive SARS-CoV-2 PCR test to delivery in X-axis. Panel C, D scatterplots show the distribution of cord blood SARS-CoV-2 IgM and IgG levels in RFU at the time of delivery in Y-axis and days from maternal first positive SARS-CoV-2 PCR test to delivery in X-axis. The different colors represent the severity of the maternal symptoms at the time of diagnosis.

338x190mm (96 x 96 DPI)

Figure 3

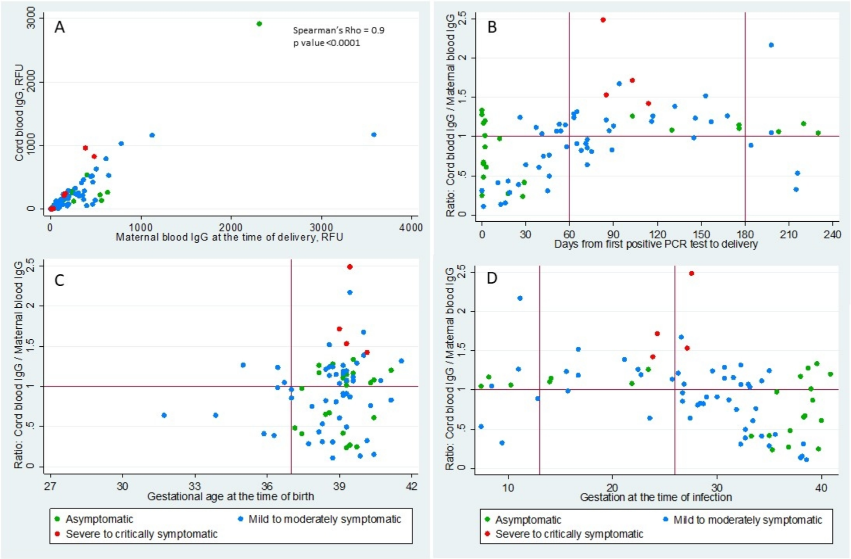

Figure 3: Correlation of cord blood and maternal IgG and distribution of IgG transplacental transfer ratio Panel A. Scatterplot shows the correlation of cord blood SARS-CoV-2 IgG levels in Y-axis and maternal blood SARS-CoV-2 IgG levels in X-axis in relative fluorescent unit (RFU). Panel B. Scatterplot shows the distribution of IgG transplacental ratio (cord blood/maternal blood SARS-CoV-2 IgG levels) in the Y-axis and days from maternal first positive SARS-CoV-2 PCR test to delivery in X-axis. Panel C. Scatterplot shows the distribution of IgG transplacental ratio in the Y-axis and gestational age at the time of delivery in X-axis. Panel D. Scatterplot shows the distribution of IgG transplacental ratio in the Y-axis and gestational age at the time of maternal first positive SARS-CoV-2 PCR test in X-axis. The different colors represent the severity of the maternal symptoms at the time of diagnosis.

338x190mm (96 x 96 DPI)

A.

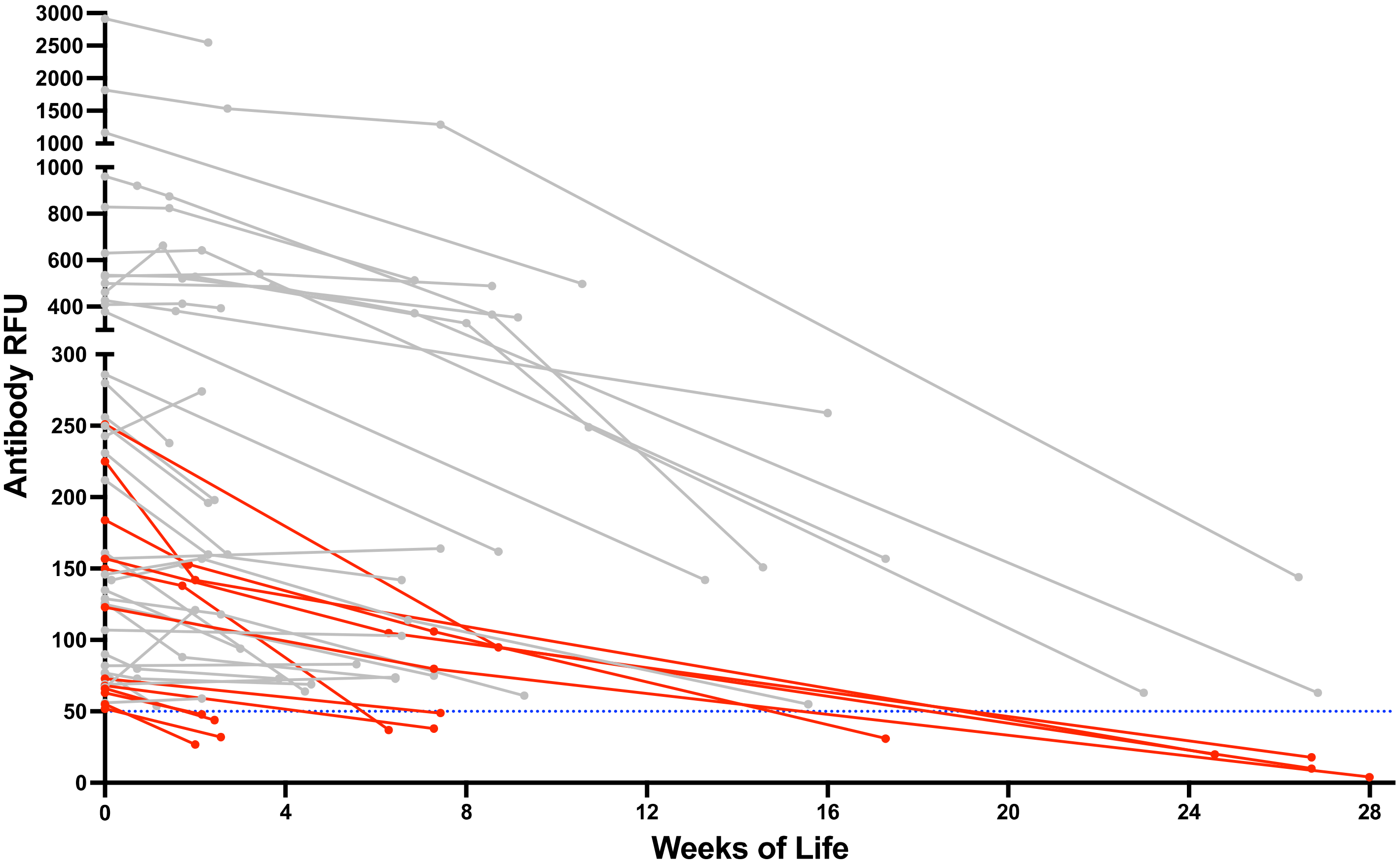
3000
2500
2000
1500
1000
1000
800
600
400
300
250
200
150
100
50
0
Antibody RFU
0
4
8
12
16
20
24
28
Weeks of Life

B.

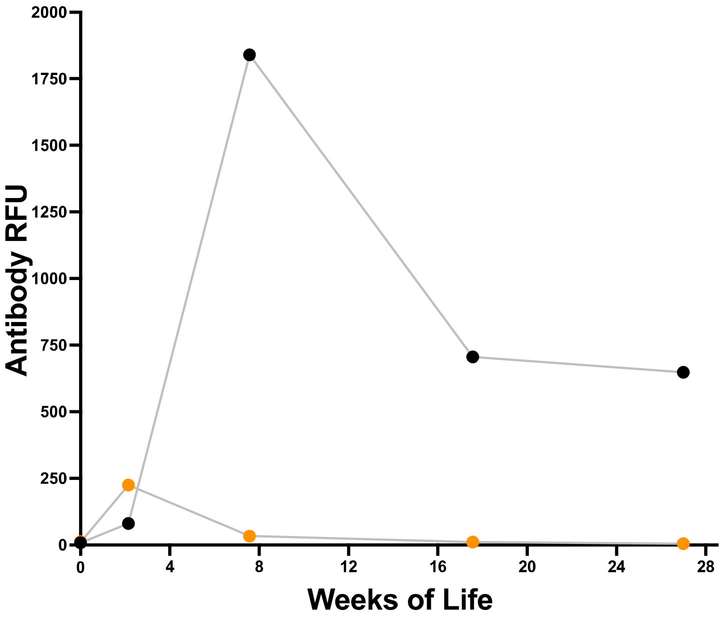
2000
1750
1500
1250
1000
750
500
250
0
Antibody RFU
0
4
8
12
16
20
24
28
Weeks of Life

C.

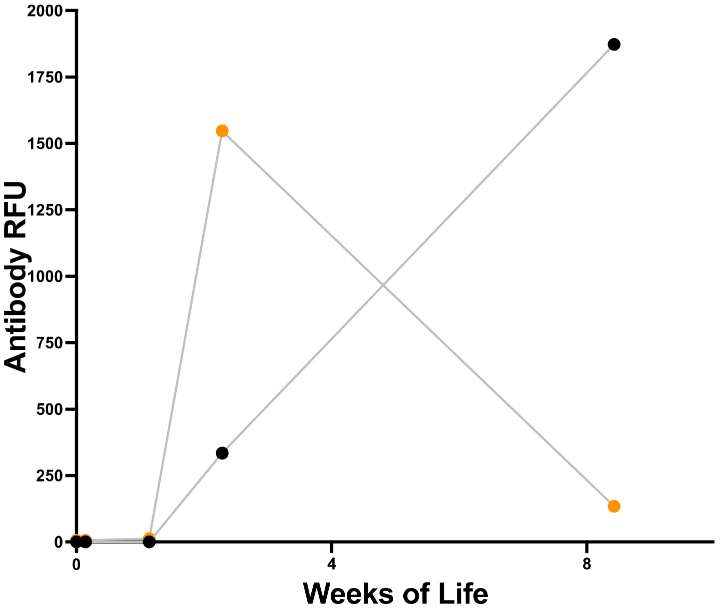
2000
1750
1500
1250
1000
750
500
250
0
Antibody RFU
0
4
8
Weeks of Life
IgG
IgM

PCR Methods:

RNA Extraction. Maternal blood, cord blood, placental tissue, and infant meconium RNA was extracted using the QIAmp Viral RNA Mini Kit following the manufacturer's instructions with some adjustments. 300uL of maternal and cord blood in RNAlater (1:1.3 ratio) were used for each extraction. 15-25 mg of placenta and 300 µg of meconium in viral transport media was used for extraction. The kit protocol was followed with buffer amounts scaled up proportionally for the starting amount. RNA was eluted in a 40uL elution buffer for blood and 20uL elution buffer for placenta and meconium. RNA quantity was measured using the Qubit RNA High Sensitivity Assay Kit.

Quantitative real-time PCR. Quantitative polymerase chain reaction was performed using the ABI StepOne Plus system. Primer sequences targeted the N (nucleotide) and Orf1b (ORF1b-nsp14) gene. Primer sequences are as follows: forward primer targeting N gene \ (HKU-NF): 5'-TAATCAGACAAGGAACTGATTA-3';Reverse primer (HKU-NR): 5'-CGAAGGTGTGACTTCCATG-3'; and Probe (HKU-NP): 5'-FAM-GCAAATTGTGCAATTTGCGG-TAMRA-3'. Forward primer targeting Orf1b-nsp14 gene (HKU-ORF1b-nsp14F): 5'-TGGGGYTTTACRGGTAACCT-3'; Reverse primer (HKU- ORF1b-nsp14R): 5'-AACRCGCTTAACAAAGCACTC-3'; and Probe (HKU-ORF1b-nsp141P): 5'-FAM-TAGTTGTGATGCWATCATGACTAG-TAMRA-3. RT-qPCR reactions were performed using the TaqMan Fast Virus 1-step Master Mix according to the manufacturer's instructions.

Table 1: PCR Reagents

Reagent	Volume per rxn (µL)
Water (RNAse free)	7.5
TaqMan Fast Virus 1-step (4X)	5
Forward Primer (10 µM)	1
Reverse Primer (10 µM)	1
Prove (10 µM)	0.5
RNA Sample	5

Table 2: PCR Cycle

Steps	Temperature (C)	Time (mm:ss) # cycles
Reverse Transcription	50	05:00
RT Inactivation/denaturation	96	00:20
Amplification	95	00:05:40
Amplification	60	00:30

Table 3: Distribution of severity of maternal symptoms at the time of diagnosis

	Asymptomatic mothers	Mild-moderately symptomatic mothers	Severe-critically symptomatic mothers
	**59**	**78**	**8**
**Time between maternal infection and delivery**			
<60 days, n	50	46	3
60-180 days, n	6	26	5
>180 days, n	3	6	0
**Trimester at the time of maternal infection**			
First Trimester, n	3	8	0
Second Trimester, n	7	12	0
Third Trimester, n	49	58	8
**Trimester at the time of delivery**			

For peer review only

First Trimester, n	0	0	0
Second Trimester, n	0	0	0
Third Trimester, n	59	78	8

Table 4: Delivery specimen PCR results

Participant	Maternal Blood	Cord Blood	Placenta	Infant Meconium
#1, cord IgM 62 RFU	Negative	Negative	Negative	-
#2, cord IgM 65 RFU	-	Negative	Negative	-
#3, cord IgM 136 RFU	-	-	Negative	Negative

For peer review only

STROBE 2007 (v4) checklist of items to be included in reports of observational studies in epidemiology*
Checklist for cohort, case-control, and cross-sectional studies (combined)

Section/Topic	Item #	Recommendation	Reported on page #
**Title and abstract**	1	(*a*) Indicate the study's design with a commonly used term in the title or the abstract	1,3,8
		(*b*) Provide in the abstract an informative and balanced summary of what was done and what was found	3
**Introduction**			
Background/rationale	2	Explain the scientific background and rationale for the investigation being reported	7
Objectives	3	State specific objectives, including any pre-specified hypotheses	7
**Methods**			
Study design	4	Present key elements of study design early in the paper	8
Setting	5	Describe the setting, locations, and relevant dates, including periods of recruitment, exposure, follow-up, and data collection	8,9
Participants	6	(*a*) *Cohort study*—Give the eligibility criteria, and the sources and methods of selection of participants. Describe methods of follow-up<br>*Case-control study*—Give the eligibility criteria, and the sources and methods of case ascertainment and control selection. Give the rationale for the choice of cases and controls<br>*Cross-sectional study*—Give the eligibility criteria, and the sources and methods of selection of participants	8,9, Figure 1
		(*b*) *Cohort study*—For matched studies, give matching criteria and number of exposed and unexposed<br>*Case-control study*—For matched studies, give matching criteria and the number of controls per case	
Variables	7	Clearly define all outcomes, exposures, predictors, potential confounders, and effect modifiers. Give diagnostic criteria, if applicable	8,9
Data sources/ measurement	8*	For each variable of interest, give sources of data and details of methods of assessment (measurement). Describe comparability of assessment methods if there is more than one group	8,9, supplemental Methods, supplemental Tables 1 and 2
Bias	9	Describe any efforts to address potential sources of bias	11,15
Study size	10	Explain how the study size was arrived at	Figure 1, 8
Quantitative variables	11	Explain how quantitative variables were handled in the analyses. If applicable, describe which groupings were chosen and why	9
Statistical methods	12	(*a*) Describe all statistical methods, including those used to control for confounding	9
		(*b*) Describe any methods used to examine subgroups and interactions	9

For peer review only - http://bmjopen.bmj.com/site/about/guidelines.xhtml

		(*c*) Explain how missing data were addressed	11
		(*d*) *Cohort study*—If applicable, explain how loss to follow-up was addressed<br>*Case-control study*—If applicable, explain how matching of cases and controls was addressed<br>*Cross-sectional study*—If applicable, describe analytical methods taking account of sampling strategy	11
		(*e*) Describe any sensitivity analyses	
**Results**			
Participants	13*	(a) Report numbers of individuals at each stage of study—eg numbers potentially eligible, examined for eligibility, confirmed eligible, included in the study, completing follow-up, and analysed	Figure 1, 9, 10,11,12,
		(b) Give reasons for non-participation at each stage	Figure 1
		(c) Consider use of a flow diagram	Figure 1
Descriptive data	14*	(a) Give characteristics of study participants (eg demographic, clinical, social) and information on exposures and potential confounders	9, Figure 2
		(b) Indicate number of participants with missing data for each variable of interest	Figure 1
		(c) *Cohort study*—Summarise follow-up time (eg, average and total amount)	8, 11
Outcome data	15*	*Cohort study*—Report numbers of outcome events or summary measures over time	Figure 1, 9, 10,11,12,
		Case-control study—Report numbers in each exposure category, or summary measures of exposure	
		Cross-sectional study—Report numbers of outcome events or summary measures	
Main results	16	(*a*) Give unadjusted estimates and, if applicable, confounder-adjusted estimates and their precision (eg, 95% confidence interval). Make clear which confounders were adjusted for and why they were included	10,11
		(*b*) Report category boundaries when continuous variables were categorized	Table 2, 10, 11
		(*c*) If relevant, consider translating estimates of relative risk into absolute risk for a meaningful time period	
Other analyses	17	Report other analyses done—eg analyses of subgroups and interactions, and sensitivity analyses	Table 2, 10, 11
**Discussion**			
Key results	18	Summarise key results with reference to study objectives	13
Limitations	19	Discuss limitations of the study, taking into account sources of potential bias or imprecision. Discuss both direction and magnitude of any potential bias	15
Interpretation	20	Give a cautious overall interpretation of results considering objectives, limitations, multiplicity of analyses, results from similar studies, and other relevant evidence	15,16
Generalisability	21	Discuss the generalisability (external validity) of the study results	15
**Other information**			
Funding	22	Give the source of funding and the role of the funders for the present study and, if applicable, for the original study on which the present article is based	4

*Give information separately for cases and controls in case-control studies and, if applicable, for exposed and unexposed groups in cohort and cross-sectional studies.

For peer review only

Note: An Explanation and Elaboration article discusses each checklist item and gives methodological background and published examples of transparent reporting. The STROBE checklist is best used in conjunction with this article (freely available on the Web sites of PLoS Medicine at http://www.plosmedicine.org/, Annals of Internal Medicine at http://www.annals.org/, and Epidemiology at http://www.epidem.com/). Information on the STROBE Initiative is available at www.strobe-statement.org.

For peer review only

BMJ Open

Passive and active immunity in infants born to mothers with SARS-CoV-2 infection during pregnancy: Prospective cohort study

Journal:	*BMJ Open*
Manuscript ID	bmjopen-2021-053036.R1
Article Type:	Original research
Date Submitted by the Author:	08-Jun-2021
Complete List of Authors:	Song, Dongli; Santa Clara Valley Health and Hospital System, Pediatrics, NICU; Stanford University School of Medicine, Deparment of Pediatrics<br>Prahl, Mary; University of California San Francisco, Department Pediatrics, Division of Pediatric Infectious Diseases and Global Health<br>Gaw, Stephanie; University of California San Francisco, Division of Maternal-Fetal Medicine, Department of Obstetrics, Gynecology and Reproductive Sciences<br>Narasimhan, Sudha Rani; Santa Clara Valley Medical Center, Department of Pediatrics, Division of Neonatology; Stanford University School of Medicine, Deparment of Pediatrics<br>Rai, Daljeet; Stanford University School of Medicine, Department of Family Medicine<br>Huang, Angela; Santa Clara Valley Medical Center, Department of Pediatrics, Division of Neonatology<br>Flores, Claudia; Santa Clara Valley Medical Center, Department of Pediatrics, Division of Neonatology<br>Lin, Christine; University of California San Francisco, Division of Maternal-Fetal Medicine, Department of Obstetrics, Gynecology and Reproductive Sciences<br>Jigmeddagva, Unurzul; University of California San Francisco, Division of Maternal-Fetal Medicine, Department of OBstetrics, Gynceology and Reproduction Sciences<br>Wu, Alan; University of California San Francisco, Department of Laboratory Medicine<br>Warrier, Lakshmi; University of California San Francisco, Department of Medicine<br>Levan, Justine; University of California San Francisco, Department of Medicine<br>Nguyen, Catherine; University of California San Francisco, Department of Medicine<br>Callaway, Perri; University of California San Francisco, Department of Medicine<br>Farrington, Lila ; University of California San Francisco, Department of Medicine<br>Acevedo, Gonzalo; University of California San Francisco, Department of Medicine<br>Gonzalez, Veronica ; University of California San Francisco, Division of Maternal-Fetal Medicine, Department of OBstetrics, Gynecology and Reproductive Sciences

	Vaaben , Anna ; University of California San Francisco, Department of Medicine<br>Nguyen, Phuong; Santa Clara Valley Medical Center, Department of Pathology<br>Atmosfera , Elda ; Santa Clara Valley Medical Center, Department of Pathology<br>Marleau, Constance ; Santa Clara Valley Medical Center, Department of Pathology<br>Anderson, Christina; Santa Clara Valley Medical Center, Department of Pediatrics, Division of Neonatology ; Stanford University School of Medicine, Department of Pediatrics<br>Misra , Sonya; Santa Clara Valley Medical Center, Department of Pediatrics, Divison of Neonatology ; Stanford University School of Medicine, Department of Pediatrics<br>Stemmle, Monica ; Santa Clara Valley Medical Center, Department of Pediatrics, Division of Neonatology ; Stanford University School of Medicine, Department of Pediatrics<br>Cortes, Maria ; Santa Clara Valley Medical Center, Departmetn of Pediatrics, Divsion of Neonatology<br>McAuley, Jennifer ; Santa Clara Valley Medical Center, Department of Pediatrics, Divison of Neonatology<br>Metz , Nicole ; Santa Clara Valley Medical Center, Department of Pediatrics, Division of Neonatology<br>Patel , Rupalee; Santa Clara Valley Medical Center, Department of Pediatrics, Division of Neonatology<br>Nudelman, Matthew; Marshall University, Department of Pediatrics<br>Abraham, Susan ; Santa Clara Valley Medical Center, Department of Pediatrics, Division of Neonatology ; Stanford University School of Medicine, Department of Pediatrics<br>Byrne , James ; Santa Clara Valley Medical Center, Department of Obstetrics and Gynecology, Division of Maternal-Fetal Medicine ; Stanford University School of Medicine, Department of Obstetrics and Gynecology<br>Jegatheesan, Priya; Santa Clara Valley Health and Hospital System, Pediatrics, Neonatology; Stanford University School of Medicine, Pediatrics

**Primary Subject Heading**:	Infectious diseases
Secondary Subject Heading:	Infectious diseases, Obstetrics and gynaecology, Paediatrics
Keywords:	COVID-19, IMMUNOLOGY, NEONATOLOGY, Paediatric infectious disease & immunisation < PAEDIATRICS

SCHOLARONE™
Manuscripts

I, the Submitting Author has the right to grant and does grant on behalf of all authors of the Work (as defined in the below author licence), an exclusive licence and/or a non-exclusive licence for contributions from authors who are: i) UK Crown employees; ii) where BMJ has agreed a CC-BY licence shall apply, and/or iii) in accordance with the terms applicable for US Federal Government officers or employees acting as part of their official duties; on a worldwide, perpetual, irrevocable, royalty-free basis to BMJ Publishing Group Ltd ("BMJ") its licensees and where the relevant Journal is co-owned by BMJ to the co-owners of the Journal, to publish the Work in this journal and any other BMJ products and to exploit all rights, as set out in our licence.

The Submitting Author accepts and understands that any supply made under these terms is made by BMJ to the Submitting Author unless you are acting as an employee on behalf of your employer or a postgraduate student of an affiliated institution which is paying any applicable article publishing charge ("APC") for Open Access articles. Where the Submitting Author wishes to make the Work available on an Open Access basis (and intends to pay the relevant APC), the terms of reuse of such Open Access shall be governed by a Creative Commons licence – details of these licences and which Creative Commons licence will apply to this Work are set out in our licence referred to above.

Other than as permitted in any relevant BMJ Author's Self Archiving Policies, I confirm this Work has not been accepted for publication elsewhere, is not being considered for publication elsewhere and does not duplicate material already published. I confirm all authors consent to publication of this Work and authorise the granting of this licence.

For peer review only

Passive and active immunity in infants born to mothers with SARS-CoV-2 infection during pregnancy: Prospective cohort study

Dongli Song, MD[1,2], Mary Prahl, MD[3], Stephanie L. Gaw, MD[4], SudhaRani Narasimhan, MD[1,2], Daljeet Rai, MD[5], Angela Huang, MPH[1], Claudia Flores, BA[1], Christine Y. Lin[4], Unurzul Jigmeddagva, MD[4], Alan H.B. Wu, PhD[6], Lakshmi Warrier[7], Justine Levan, PhD[7], Catherine B.T. Nguyen[7], Perri Callaway[7], Lila Farrington, PhD[7], Gonzalo R. Acevedo, PhD[7], Veronica J. Gonzalez, MD[4], Anna Vaaben, MS[7], Phuong Nguyen, MBA[8], Elda Atmosfera, CLS[8], Constance Marleau, MT[8], Christina Anderson, MD[1,2], Sonya Misra, MD[1,2], Monica Stemmle, MD[1,2], Maria Cortes, DNP[1], Jennifer McAuley, NNP[1], Nicole Metz, BSN[1], Rupalee Patel, DNP[1], Matthew Nudelman[9], Susan Abraham, MD[1,2], James Byrne, MD[10. 11], Priya Jegatheesan, MD[1,2*]

[1]Department of Pediatrics, Division of Neonatology, Santa Clara Valley Medical Center, San Jose, CA, USA

[2]Department of Pediatrics, Stanford University School of Medicine, Stanford, CA, USA

[3]Department of Pediatrics, Division of Pediatric Infectious Diseases and Global Health, University of California, San Francisco, CA, USA

[4]Division of Maternal-Fetal Medicine, Department of Obstetrics, Gynecology and Reproductive Sciences, University of California, San Francisco, CA, USA

[5]Department of Family Medicine, Stanford University School of Medicine, Stanford, CA, USA

[6] Department of Laboratory Medicine, University of California, San Francisco, CA, USA

[7] Department of Medicine, University of California San Francisco, CA, USA

[8]Department of Pathology, Santa Clara Valley Medical Center, San Jose, CA, USA

[9]Department of Pediatrics, Marshall University, Huntington, WV, USA

[10]Department of Obstetrics and Gynecology, Division of Maternal-Fetal Medicine, Santa Clara Valley, CA, USA

[11]Department of Obstetrics and Gynecology, Stanford University School of Medicine, Stanford, CA, USA

For peer review only

Corresponding Author

Priya Jegatheesan, MD

751 South Bascom Ave, San Jose, CA 95128

Email: Priya.jegatheesan@hhs.sccgov.org

Phone: Mobile 408-687-3493 Office 408-885-5420

For peer review only

Abstract

OBJECTIVE To investigate maternal immunoglobulins' (IgM, IgG) response to SARS-CoV-2 infection during pregnancy and IgG transplacental transfer, to characterize neonatal antibody response to SARS-CoV-2 infection, and to longitudinally follow actively- and passively-acquired antibodies in infants.

DESIGN A prospective observational study.

SETTING Public healthcare system in Santa Clara County (CA, USA).

PARTICIPANTS Women with symptomatic or asymptomatic SARS-CoV-2 infection during pregnancy and their infants were enrolled between April 15, 2020 and March 31, 2021.

OUTCOMES SARS-CoV-2 serology analyses in the cord and maternal blood at delivery and longitudinally in infant blood between birth and 28 weeks of life.

RESULTS Of 145 mothers who tested positive for SARS-CoV-2 during pregnancy, 86 had symptomatic infections: 78 with mild-moderate symptoms, and eight with severe-critical symptoms. The seropositivity rates of the mothers at delivery was 65% (95% CI 0.56-0.73) and the cord blood was 58% (95% CI 0.49-0.66). IgG levels significantly correlated between the maternal and cord blood (Rs= 0.93, p< 0.0001). IgG transplacental transfer ratio was significantly higher when the first maternal positive PCR was 60-180 days before delivery compared to <60 days (1.2 vs. 0.6, p=<0.0001). Infant IgG seroreversion rate over follow-up periods of 1-4, 5-12, and 13-28 weeks were 8% (4/48), 12% (3/25), and 38% (5/13), respectively. The IgG seropositivity in the infants was positively related to IgG levels in the cord blood and persisted up to six months of age. Of the 147 newborns, two infants showed seroconversion at two weeks of age with high levels of IgM and IgG, including one premature infant with confirmed intrapartum infection.

CONCLUSIONS Maternal SARS-CoV-2 IgG is efficiently transferred across the placenta when infections occur more than two months before delivery. Maternally-derived passive immunity may persist in infants up to six months of life. Two neonates mounted a strong antibody response to perinatal SARS-CoV-2 infection.

For peer review only

Strengths and limitations of this study

- This study included pregnant mothers with SARS-CoV-2 infection in all three trimesters of pregnancy and provided a comprehensive understanding of maternal SARS-CoV-2 IgG transplacental transfer throughout pregnancy.
- This is the first longitudinal study that has followed maternally-derived SARS-CoV-2 IgG in infants up to 28 weeks.
- This is the first study, to our knowledge, that characterized neonatal serology response to perinatal SARS-CoV-2 in two neonates.
- In asymptomatic mothers who were identified as SARS-CoV-2 PCR positive at the time of delivery, we were unable to ascertain the precise timing of infection.
- The cohort had few severe cases of maternal infection and premature births before 35 weeks of gestation.

Funding statement

This works was supported by Bill and Melinda Gates Foundation (INV-017035 to SLG), National Institutes of Health (NIAID K08AI141728 to SLG, NIAID K23AI127886 to MP), Marino Family Foundation, and Valley Medical Center Foundation. The funders did not have any role in the study design, patient recruitment, data collection, analysis, or interpretation of the results. All the authors had full access to the full data in the study and accept responsibility to submit for publication.

Competing interests' statement

None declared.

Author contributions

For peer review only

D.S. conceptualized and designed the project, participated in patient enrollment, sample collection, data visualization and interpretation, and wrote the manuscript draft.

P.J. conceptualized and designed the project, participated in patient enrollment, sample collection, data collection, analysis, interpretation, and edited manuscript.

M.P., S.L.G designed collection and processing protocols, performed sample processing, oversaw experiments and data analysis, provided funding, edited the manuscript.

S.R.N. conceptualized and designed the project, participated in patient enrollment, sample collection, data collection and edited manuscript.

D.R. participated in study design, patient enrollment, and sample collection.

A.H. coordinated data collection and management, participated in sample collection, and oversaw the implementation of the project.

C.V.F. participated in patient enrollment, coordinated sample collections, processing, and data management

L.W. designed collection and processing protocols, performed sample processing and data collection.

J.L. and C.B.T.N., CYL, UJ, VJG designed and performed experiments, performed sample processing and data collection.

P.C., L.F, G.R.A., A.V. optimized collection and processing protocols, performed sample processing and data collection

A.H.B.W. designed and oversaw serology assays.

E.A., P.N., C.M. oversaw sample collections and processing.

C.A., S.M., M.S., M.C., J.M., S.A., N.M. participated in patient enrollment and sample collection

M.N. participated in data analysis and preparing the figures for the manuscript.

R.P. participated in the study implementation, and sample collection.

J.B. designed, and oversaw the patient recruitment and implementation of the project

All authors reviewed and approved the manuscript.

For peer review only

Data sharing statement:

De-identified data is published in Mendeley data sharing site and available in the following doi:10.17632/6scfwt55fd.2.

Word count

3955 words

For peer review only

Introduction

Our understanding of the immune response to severe acute respiratory syndrome coronavirus-2 (SARS-CoV-2) is expanding rapidly through extensive basic and clinical studies.[1-4] However, the literature on SARS-CoV-2 immunity in pregnant mothers and infants remains limited.[5-9] Global efforts are focused on controlling the COVID-19 pandemic through public health prevention measures and universal vaccination. Knowledge of neonatal immune response to SARS-CoV-2 and maternally-derived passive immunity in young infants is urgently needed to inform ongoing COVID-19 infection prevention and vaccination strategies to protect pregnant mothers and infants.

The physiological changes occurring during pregnancy make the mothers more vulnerable to severe respiratory infections. The Centers for Disease Control (CDC) reported that Covid-19 infection poses a significantly higher risk for severe illness and death in symptomatic infected pregnant than symptomatic infected nonpregnant women.[10] An international study collected outcomes of 706 pregnant mothers with SARS-CoV-2 infection during pregnancy and their newborns from 18 developed and developing counties.[11] Results from this large-scale study demonstrated that pregnant women with COVID-19, compared with those without COVID-19, were at a substantially increased risk of severe pregnancy complications and death. Interestingly, several cohort studies conducted in the US have found that the majority of pregnant women with SARS-CoV-2 infection were either asymptomatic or had mild symptoms.[5,12-16]

Neonatal infection following birth to a mother with SARS-CoV-2 infection during pregnancy is infrequent.[16-21] CDC reported that the perinatal SARS-CoV-2 infection rate among infants born to mothers with COVID-19 during pregnancy was 2.6%.[17] Notably, the majority of the infected infants were born to mothers who had infections within one week of delivery. A meta-analysis review of 174 neonatal infection cases found that 70% and 30% of infections are due to environmental and vertical transmission, respectively. Fifty-five percent of infected neonates were symptomatic, including fever (44%),

For peer review only

gastrointestinal (36%), respiratory (52%), and neurological manifestations (18%).[22] Data from CDC and other case studies showed that the majority of infected neonates were asymptomatic or exbibit mild symptoms.[16,17,23] However, in a UK national population-based cohort study, 42% of the infected infants presented with severe symptoms.[20,24] The large-scale international investigation found that infants born to women with COVID-19 during pregnancy had a significantly higher risk for severe perinatal mobility and mortality.[11] These risks remained significant after adjusting for prematurity, indicating a direct effect of COVID-19 on the infants.

Children are more vulnerable to severe respiratory infections. Interestingly, children, compared with adults, are less susceptible to SARS-CoV-2 infection and less likely to develop severe illness.[25,26] However, children with certain underlying medical conditions and infants (age <1 year) might be at increased risk for severe illness from SARS-CoV-2 infection.[27,28] A study from China included Chinese children with confirmed and suspected SARS-CoV-2 infection.[28] Among them, 18% were less than one year old, and 10% of the infants had severe or critical clinical symptoms. In a small case series from the US, all 18 less than three-month-old infants with COVID-19 presented with mild symptoms.[29]

An important aspect of immunity against infectious pathogens in young infants relies on effective maternal antibody production, transfer of maternal antibodies across the placenta to the fetus, and persistence of passive immunity in the infant. Recent publications have shown evidence of maternal SARS-CoV-2 antibody transplacental transfer.[6,7,9] However, the majority of maternal SARS-CoV-2 infections in these reports occurred late in pregnancy, as these studies were conducted during the first few months of the COVID-19 pandemic. Therefore, the timing and efficiency of maternal antibody production and transplacental transfer throughout gestation remain to be fully understood, which has important implications for the timing of maternal immunization to benefit both pregnant mothers and their young infants. Furthermore, the important question as to the persistence of maternally-derived passive immunity in infants needs to be investigated. While SARS-CoV-2 infection has been described in newborns,[17,22]

For peer review only

little is known about infant immune response to perinatal infection. The aims of this study were to investigate SARS-CoV-2 antibody transplacental transfer with respect to the timing of maternal infection during gestation, antibody response to SARS-CoV-2 infection in the newborns, and persistence of passively- and actively-acquired SARS-CoV-2 antibodies in infants.

Methods:

Study design, participants, and procedures

This is a prospective observational study of pregnant mothers with SARS-CoV-2 infection during pregnancy and their infants. The study was conducted from April 15, 2020 to March 31, 2021, in a public healthcare system, including one regional medical center and two community hospitals. The healthcare system primarily serves the medically indigent population of Santa Clara County California (USA). The study obtained ethics approval from the institutional review board of Santa Clara Valley Medical Center, IRB reference # 20-021. Patients provided written informed consent prior to study enrollment and all study procedures.

On April 15th, 2020, our institution implemented universal SARS-CoV-2 screening protocol in Labor and Delivery units. All women who were admitted for delivery or within three days prior to admission for elective deliveries were tested for SARS-CoV-2 by PCR using a nasopharyngeal swab.[30] From October 2020 onwards, women who tested positive within 90 days prior to admission for delivery and did not have new symptoms of COVID were not retested at the time of delivery. In addition to PCR testing, mothers were screened for history of SARS CoV-2 infection and PCR testing during pregnancy. PCR tests were done anytime during pregnancy if the mother experienced symptoms concerning for COVID-19 or had close contact with a person with COVID-19. The pregnant mothers who had a documented positive SARS-CoV-2 PCR during the current pregnancy, either prior to admission or tested positive after admission, were eligible for the study. We screened and enrolled mothers for the study after they were admitted to the Labor and Delivery units. The timing of maternal SARS-CoV-2 infection was based on

For peer review only - http://bmjopen.bmj.com/site/about/guidelines.xhtml

the first positive SARS-CoV-2 PCR test. The severity of SARS-CoV-2 symptoms (mild, moderate, severe, or critical) was assessed according to the Society for Maternal-Fetal Medicine guidelines.[31]

If the maternal infection was within 10-14 days of delivery, the mother and infant roomed in together with airborne isolation precautions and the mother wore a surgical mask when holding and breastfeeding the baby during the isolation period. The nasopharyngeal SARS-CoV-2 PCR was performed in the newborns at 24 hours of life. The infants were retested between 48-72 hours of life if they were in the Neonatal Intensive Care Unit (NICU). Maternal and neonatal nasopharyngeal samples were collected according to hospital standard procedure. PCR tests were performed by hospital clinical laboratories using validated SARS-CoV-2 assays for clinical diagnosis (supplemental Methods).

Maternal and cord blood were collected at the time of delivery. Serial infant blood samples were initially designed to be collected at two weeks, two months, and six months coordinated with routine pediatric clinic visits. During the pandemic the visit schedules varied significantly due to parental hesitance to come to the clinics for concerns of COVID exposure. Thus, infants' blood samples were collected anytime between 1-4 weeks, 5-12 weeks, and 13-28 weeks at the time of clinic visits. Levels of SARS-CoV-2 immunoglobulin M (IgM) and immunoglobulin G (IgG) to the spike protein receptor binding domain (RBD) and nucleocapsid protein (NP) of SARS-CoV-2 were measured using the Pylon 3D automated immunoassay system (ET Healthcare, Palo Alto, CA) as previously described.[32] The background corrected signal was reported as relative fluorescent units (RFU), which is proportional to the amount of specific antibodies in the sample allowing for quantification. The positive cutoffs for IgM and IgG were set to >50 RFU to achieve 100% specificity and a high level of sensitivity.[32] Quantitative reverse transcriptase PCR (qRT-PCR) was performed on maternal blood, cord blood, placenta, and meconium in a subset of infants. Primer sequences targeted the N and Orf1b SARS-CoV-2 genes (supplemental Methods, supplemental Tables 1 and 2).

For peer review only

Data collection and analysis

Clinical data included maternal and neonatal demographics, the severity of maternal symptoms of SARS-CoV-2 infection, days between maternal first positive SARS-CoV-2 PCR test and delivery, and neonatal outcomes. Demographics, clinical outcomes, and serum IgM and IgG levels were summarized using descriptive analyses. Transplacental IgG transfer ratios were calculated by dividing cord blood IgG levels by maternal blood IgG levels. Correlation between maternal and cord blood IgG levels and correlation between placental transfer ratio and gestational age (GA) at birth were analyzed using Spearman's rank-order correlation. The transfer ratios were compared between maternal groups based on infection severity and time between first maternal positive PCR and delivery using the Kruskal-Wallis test, followed by Dunn's test for pairwise multiple comparisons with the Holm-Sidák stepwise adjustment.

Patient and Public involvement

Patients or the public were not involved in the design, or conduct, or reporting, or dissemination plans of our research

Results

During the study period, 3936 mothers delivered in the health system with 3956 live births, and 254 (6.5%) of the mothers had at least one positive SARS-CoV-2 PCR test during the pregnancy. The study enrolled 145 mothers with SARS-CoV-2 infection and 147 of their infants (Figure 1). Of 145 enrolled mothers, 86 (59%) had symptomatic infection, including 78 with mild-moderate symptoms and eight with severe-critical symptoms (Table 1). The distribution of the severity of the maternal infection is shown in supplemental Table 3. Of 147 newborns, 23 (16%) were admitted to the NICU. SARS-CoV-2 PCR was performed on nasopharyngeal specimens of 89 newborns at 24 hours of life, and only one 31-week preterm infant tested positive.

Maternal and cord blood serology

For peer review only

Serum serology was performed on 129 mothers at delivery and 144 cord blood samples. The temporal profiles of maternal blood IgM and IgG with respect to the timing of first maternal PCR positivity are shown in Figure 2A and Figure 2B. Antibody status and levels in maternal and cord blood were evaluated in four groups based on the days between maternal first positive SARS-CoV-2 PCR and delivery (<14 days, 14 to 59 days, 60 to 180 days, and >180 days) (Table 2). The maternal IgG level at the time of delivery was significantly lower in <14 days group compared to 14-59 days, 60-180 days, and >180 days (p=0.0001) (Figure 2C, Table 2). The overall maternal seropositivity rate at delivery was 65% (84/129, 95% CI 0.56-0.73). Of the 31 mother who were asymptomatic and identified by positive SARS-CoV-2 at delivery, ten had serology tests positive for IgG but negative for IgM, consistent with convalescent infections. The temporal profile of cord blood IgG with respect to the timing of first maternal PCR positivity is shown in Figure 2D. The cord blood IgG positivity rate was 58% (83/144, 95% CI 0.49-0.66).

Paired serology analysis was performed in 125 maternal-cord blood samples (Table 2). Of the 77 IgG positive mothers, 69 (90%) of their newborns' cord sera were positive for IgG. Of the eight IgG negative infants, seven were born to mothers with infection within 45 days of delivery, and one was born to a mother who had a positive PCR at 254 days before delivery. Of the 48 IgG negative mothers, 45 (94%) of their newborns' cord sera were negative for IgG. Of the 125 cord samples, there were three infants whose cord blood was positive for IgM (65, 136, and 62 RFU). Notably, all three were born to mothers whose blood was also positive for IgM at the time of delivery. The follow-up serology tests for two of the infants at two and three weeks of age were negative for IgM and IgG. No follow-up serology was available for the third infant. Available delivery specimens (maternal and cord blood, placenta, and meconium) were evaluated by SARS-CoV-2 PCR and found to be negative for all three infants (supplemental Table 4). Two of these were term infants and had a normal newborn course in the hospital and remained asymptomatic during the first month of life. The third infant was a 31 weeks gestational age premature infant who was delivered due to in utero growth restriction. This infant had typical respiratory symptoms

For peer review only

due to lung immaturity. The chest X ray did not show any evidence of infiltration. The infant was on CPAP and nasal canula, with 21% FiO2 for three weeks.

There was a significant positive correlation between IgG levels in the 125 paired maternal and cord blood samples (Rs=0.93, p<0·0001, Figure 3A). Transplacental IgG transfer ratios were calculated in 77 IgG positive mother-infant dyads, and the median transfer ratio was 1.0 (95% CI 0.86-1.09). The transfer ratio was significantly higher in the mothers who were severe-critically symptomatic (1.6, 95% CI 1.42-2.49, n=4) compared to mothers who were asymptomatic (1.0, 95% CI 0.62-1.14, n=23) (1.6 vs. 1.0, p=0.003) or mild-moderately symptomatic (0.9, (95% CI 0.81-1.09, n=50) (1.6 vs. 0.9, p=0.002). To illustrate the temporal effect of maternal infection on transfer efficiency, we analyzed transfer ratios of 54 symptomatic mother-infant dyads. Asymptomatic mothers were excluded from this analysis as their timing of infections cannot be concluded definitively from the timing of PCR positivity (Figure 3B). The transfer ratios based on time elapsed from the first maternal positive PCR to delivery were 0.6 (95% CI 0.39-1.04) (<60 days, n=22), 1.2 (95% CI 0.98-1.29) (60-180 days, n=27), and 0.9 (95% CI 0.33-2.17) (>180 days, n=5). The ratio was significantly higher in the 60-180 days group compared to the <60 days group (1.2 vs. 0.6, p=<0.0001). There was no significant correlation between the transfer ratio and GA at birth (Rs=0.18, p=0.1, Figure 3C); however, 95% of the infants in our cohort were born at greater than 34 weeks gestation. Transfer ratios based on the trimester of maternal infection were 0.9 (95% CI 0.39-1.89) (1st trimester, n=7), 1.2 (95% CI 1.08-1.5) (2nd trimester, n=9), and 0.9 (95% CI 0.76-1.1) (3rd trimester, n=38) (Figure 3D). The ratio was significantly higher in second trimester infections than third trimester infections (1.2 vs. 0.9, p=0.02).

Maternally-derived IgG longitudinal follow-up in infants

To evaluate maternally-derived IgG persistence postnatally, we followed serology in 48 infants with positive cord IgG. All infants showed a steady decrease in IgG levels over time (Figure 4A). The IgG

For peer review only

seroreversion rate was calculated for those infants who had at least one serology test during the follow-up age periods of 1-4 weeks, 5-12 weeks, and 13-28 weeks. The IgG seroreversio rates for the three follow-up periods were 8% (4/48), 12% (3/25), and 38% (5/13), respectively. The infants who had lower levels of IgG in the cord blood became IgG negative earlier; the infants who had cord IgG levels were 52-66 RFU seroreverted at 1-4 weeks, 68-150 RFU seroreverted at 5-12 weeks, and 123-251 RFU seroreverted at 13-28 weeks. Two infants who had cord IgG levels greater than 500 RFU remained seropositive at 27 weeks of age.

Infant antibody response to perinatal SARS-CoV-2 infection

We performed surveillance serology tests at 2-4 weeks of age in 23 of 41 (56%) infants who had negative serology in the cord blood and were born to mothers with first positive PCR <14 days before delivery. Two infants showed seroconversion, including the 31-week preterm infant who tested positive for SARS-CoV-2 nasopharyngeal swab and a term infant. Interestingly, both infants were born to mothers who tested positive for SARS-CoV-2 PCR for the first time at delivery and negative for SARS-CoV-2 antibodies, indicating a new onset of infection. Both mothers were asymptomatic at delivery and remained asymptomatic for two weeks post delivery. Both infants were asymptomatic for SARS-CoV-2 infection. The preterm infant, was admitted to the NICU immediately after birth, isolated from the mother for 14 days. This infant had typical respiratory symptoms for 31 weeks prematurity and was on CPAP and nasal canula, with 21% FiO2 for 10 days. The chest X ray did not show any evidence of infiltration. The infant did not have any symptoms or concerns attributable to COVID-19 disease during the NICU stay and was discharge home at 34 weeks and 5 days post menstrual age.

The infant's cord blood SARS-VoC-2 PCR was negative, but nasopharyngeal PCR was positive at 24 hours of life and remained positive at discharge. Additionally, the infant's meconium and maternal blood at the time of delivery were PCR positive. The term infant roomed in with the mother in the postpartum

For peer review only

unit and was discharged home at two days of life. This infants' cord blood and nasopharyngeal SARS-CoV-2 PCR were negative at 24 hours of life, and nasopharyngeal PCR was not repeated.

The preterm infant showed serial negative serology tests after birth on days two, four, and eight, then seroconverted on day 16 (IgM 1548 RFU, IgG 335 RFU) (Figure 4B). The infant's IgM decreased to 134 RFU, and IgG increased to 1873 RFU at eight weeks. The term infant had the first follow-up test at two weeks and was found positive for IgM (225 RFU) and IgG (80RFU) (Figure 4C). The infant's IgM became negative, and IgG peaked at 1841 RFU at eight weeks; the IgG subsequently decreased to 648 RFU at 24 weeks.

Discussion

We conducted a prospective observational study in 145 pregnant mothers with SARS-CoV-2 infections during pregnancy and 147 of their infants. The majority of infected mothers seroconverted before delivery. The IgG levels in maternal blood at delivery and cord blood were highly correlated. High transplacental IgG transfer ratios were observed when infection onset was greater than 60 days prior to delivery or in the second trimester. The persistence of maternal-derived IgG in infants was positively correlated to the initial cord blood level. Additionally, we showed strong antibody responses to perinatal SARS-CoV-2 infection in two asymptomatic neonates.

This study was conducted from April 2020 to March 2021 when Northern California experienced the peaks of COVID-19 pandemic outbreaks. During this period, our universal screening program in Labor and Delivery units identified, 6.5% of mothers who had at least one positive SARS-CoV-2 PCR during their current pregnancy. It is possible that positive cases would have been missed as some asymptomatic or mildly symptomatic mothers were not tested. The majority of mothers had asymptomatic or mild-moderate infections, consistent with previous cohort studies.[12,16] The maternal IgG levels at delivery were relatively low, comparable to levels in non-ICU patients.[32] Importantly, the temporal profiles of maternal

For peer review only

and cord blood IgG levels were in parallel, peaking around 60-120 days post maternal infection. The timing and efficiency of maternal IgG transfer have important implications for developing maternal immunization strategies to protect infants.[33-35] For example, in maternal pertussis immunization, infant seropositivity rate and cord blood IgG levels to pertussis toxin were higher following Tdap immunization during the second trimester than during the third trimester. We studied pregnant mothers who had SARS-CoV-2 infections in all three trimesters and provide a comprehensive profile of transplacental IgG transfer with respect to the timing of infections throughout gestation. We observed that transfer ratio was 0.6 when infection onset was less than 60 days before delivery; plateaus at 1.2 and 0.9 when infections occurred 60-180 days and greater than 180 days before delivery. Prior studies of pregnancy related infection in the last 70 days of gestation found impaired SARS-CoV-2 IgG transplacental transfer (ratio 0.7).[7,8] Another study characterized a cohort of pregnant mothers who had infections during the last 120 days of gestation and showed that transfer ratios increased with length of time from infection to delivery, with transfer ratios reached above 1.0 in the majority of mothers.[6] Taken together, these studies demonstrate that cross-placental SARS-CoV-2 IgG transfer occurs throughout gestation, and a higher transfer efficiency is achieved when infection onset is more than two months prior to delivery. Matching the peak IgG transplacental transfer and the peak immune response after maternal infection may result in high cord IgG. Information from these maternal and cord serology studies is helpful to inform the timing of maternal vaccination in pregnancy to optimize neonatal immunity in concert.

While the persistence of maternal-derived IgG in infants showed a wide range, from two weeks to more than 26 weeks of age, the patterns of IgG decay in these infants were very similar. An important observation is that IgG positivity in infants is positively associated with the initial cord IgG levels that are determined by maternal IgG levels and transplacental transfer efficiency. As more pregnant mothers are vaccinated for SARS-CoV-2, knowledge of passive immunity in infants may inform mother-infant care and SARS-CoV-2 vaccination strategy in infants.

For peer review only

Consistent with prior literature showing rare vertical maternal-fetal transmission,[18-21] we found only one infant with confirmed intrapartum acquired neonatal infection. (21) This infant was seronegative in cord blood and during the first week of life but seroconverted at two weeks of life, providing insight into the timing of infant seroconversion in the setting of intrapartum infection. We identified another infant who seroconverted at two weeks follow-up test; however, available virology and serology data is not sufficient to determine the timing and mode of this perinatal infection. Clinical presentations of perinatal SARS-CoV-2 infection have been described previously;[17,22 36] however, little is known about neonatal serology response and long-term clinical outcomes. Interestingly, both infants in our study had asymptomatic infection but mounted strong antibody responses; the timing of seroconversion and levels of IgM and IgG are comparable to that observed in adult patients with severe disease.[32] Both infants remained asymptomatic in the first months of life. Their long-term clinical outcomes, along with immune status, will be followed. Additionally, these two cases highlight the increased risk for perinatal SARS-CoV-2 infection in infants born to mothers who have new-onset infections around the time of delivery,[17] with implications for developing targeted protection measures and postnatal antibody screening for high-risk newborns.

In our study, three infants were positive for IgM in cord blood but negative for SARS-CoV-2 virologically in birth specimens and negative for IgM and IgG at two and three weeks of age, suggesting these transient IgM levels may be false positives or maternal blood contamination. There were two prior case reports describing similar transient positive IgM levels in the cord blood without virological evidence of infection.[37,38] Thus, diagnosis of congenital SARS-CoV-2 infection cannot be made based solely on the presence of IgM in the cord blood.[39-42]

This maternal-infant serology study, one of the largest cohorts to date, included pregnant mothers with SARS-CoV-2 infection in all three trimesters of pregnancy and provided a more comprehensive understanding of maternal SARS-CoV-2 IgG transplacental transfer. This is the first longitudinal study

For peer review only

that has followed the level of maternally-derived SARS-CoV-2 IgG in infants up to 28 weeks and neonatal serology response after perinatal SARS-CoV-2 infection up to 24 weeks. Another strength of the study is that the cohort is representative of COVID-19 in the community. Over 90% of the mothers in this cohort are Hispanic, a population highly impacted by the COVID-19 pandemic.

Our study has several limitations. It was conducted in a single healthcare system. Our cohort had few severe cases and premature births before 35 weeks of gestation. Our longitudinal infant serology follow up had significant attrition and the timing of blood sampling was variable due to the challenges of coming to the clinics during the pandemic. The timing of maternal infection was based on the first positive PCR. In asymptomatic mothers with first positive SARS-CoV-2 PCR at the time of delivery we were unable to ascertain the precise timing of infection. Universal screening at the time of admission also introduces a bias in the identification of asymptomatic SARS-CoV-2 cases at or near-term gestation, as the universal screening was not implemented in our prenatal care visits and asymptomatic screening was not readily available in our general community during the study period.

Conclusion

Our study provides insights into the intricate connections between the timing of maternal SARS-CoV-2 infection, dynamics of maternal antibody production, and transplacental immunity transfer. These processes determine the level of maternally-derived IgG in infants at birth, which in turn affects persistence of passive immunity in infants. Neonates are capable of mounting strong serology responses to perinatal SARS-CoV-2 infection. These findings have important implications in determining optimal timing of vaccination in pregnant mothers and infants. Future investigations are needed to address the durability and protection of passively and actively acquired antibodies in the infant.

Acknowledgements

Thank you to Dr. Margaret Feeney for support of these experiments and Robin D. Wu who reviewed the manuscript and provided helpful comments and suggestions. We thank the mothers, newborns and their

families who participated in the study; staff and providers in Labor and Delivery, Post-partum unit, NICU, pathology and laboratory services, at Santa Clara Valley Medical Center and O'Connor Hospital; outpatient pediatrics clinic, and BRIDGE home follow up program. We thank First 5 of Santa Clara County for their support.

References

1. Blanco-Melo D, Nilsson-Payant BE, Liu WC, et al. Imbalanced Host Response to SARS-CoV-2 Drives Development of COVID-19. *Cell.* 2020;181(5):1036-1045.e1039.

2. Lucas C, Wong P, Klein J, et al. Longitudinal analyses reveal immunological misfiring in severe COVID-19. *Nature.* 2020;584(7821):463-469.

3. Briere CE, Lucas R, McGrath JM, Lussier M, Brownell E. Establishing breastfeeding with the late preterm infant in the NICU. *J Obstet Gynecol Neonatal Nurs.* 2015;44(1):102-113.

4. Stephens DS, McElrath MJ. COVID-19 and the Path to Immunity. *JAMA.* 2020;324(13):1279-1281.

5. Flannery DD, Gouma S, Dhudasia MB, et al. SARS-CoV-2 seroprevalence among parturient women in Philadelphia. *Sci Immunol.* 2020;5(49).

6. Flannery DD, Gouma S, Dhudasia MB, et al. Assessment of Maternal and Neonatal Cord Blood SARS-CoV-2 Antibodies and Placental Transfer Ratios. *JAMA Pediatr.* 2021.

7. Edlow AG, Li JZ, Collier AY, et al. Assessment of Maternal and Neonatal SARS-CoV-2 Viral Load, Transplacental Antibody Transfer, and Placental Pathology in Pregnancies During the COVID-19 Pandemic. *JAMA Netw Open.* 2020;3(12):e2030455.

8. Atyeo C, Pullen KM, Bordt EA, et al. Compromised SARS-CoV-2-specific placental antibody transfer. *Cell.* 2021;184(3):628-642.e610.

9. Kubiak JM, Murphy EA, Yee J, et al. Severe acute respiratory syndrome coronavirus 2 serology levels in pregnant women and their neonates. *Am J Obstet Gynecol.* Epub January 23, 2021.

10. Zambrano LD, Ellington S, Strid P, et al. Update: Characteristics of Symptomatic Women of Reproductive Age with Laboratory-Confirmed SARS-CoV-2 Infection by Pregnancy Status - United States, January 22-October 3, 2020. *MMWR Morb Mortal Wkly Rep.* 2020;69(44):1641-1647.

For peer review only

11. Villar J, Ariff S, Gunier RB, et al. Maternal and Neonatal Morbidity and Mortality Among Pregnant Women With and Without COVID-19 Infection: The INTERCOVID Multinational Cohort Study. *JAMA Pediatr.* 2021.

12. Sutton D, Fuchs K, D'Alton M, Goffman D. Universal Screening for SARS-CoV-2 in Women Admitted for Delivery. *N Engl J Med.* 2020;382(22):2163-2164.

13. Handley SC, Mullin AM, Elovitz MA, et al. Changes in Preterm Birth Phenotypes and Stillbirth at 2 Philadelphia Hospitals During the SARS-CoV-2 Pandemic, March-June 2020. *JAMA.* 2021;325(1):87-89.

14. Blitz MJ, Rochelson B, Rausch AC, et al. Universal testing for coronavirus disease 2019 in pregnant women admitted for delivery: prevalence of peripartum infection and rate of asymptomatic carriers at four New York hospitals within an integrated healthcare system. *Am J Obstet Gynecol MFM.* 2020;2(3):100169.

15. Fassett MJ, Lurvey LD, Yasumura L, et al. Universal SARS-Cov-2 Screening in Women Admitted for Delivery in a Large Managed Care Organization. *Am J Perinatol.* 2020;37(11):1110-1114.

16. Adhikari EH, Moreno W, Zofkie AC, et al. Pregnancy Outcomes Among Women With and Without Severe Acute Respiratory Syndrome Coronavirus 2 Infection. *JAMA Netw Open.* 2020;3(11):e2029256.

17. Woodworth KR, Olsen EO, Neelam V, et al. Birth and Infant Outcomes Following Laboratory-Confirmed SARS-CoV-2 Infection in Pregnancy - SET-NET, 16 Jurisdictions, March 29-October 14, 2020. *MMWR Morb Mortal Wkly Rep.* 2020;69(44):1635-1640.

18. Salvatore CM, Han JY, Acker KP, et al. Neonatal management and outcomes during the COVID-19 pandemic: an observation cohort study. *Lancet Child Adolesc Health.* 2020;4(10):721-727.

19. Flaherman VJ, Afshar Y, Boscardin J, et al. Infant Outcomes Following Maternal Infection with SARS-CoV-2: First Report from the PRIORITY Study. *Clin Infect Dis.* 2020.

20. Gale C, Quigley MA, Placzek A, et al. Characteristics and outcomes of neonatal SARS-CoV-2 infection in the UK: a prospective national cohort study using active surveillance. *Lancet Child Adolesc Health.* 2021;5(2):113-121.

21. Dumitriu D, Emeruwa UN, Hanft E, et al. Outcomes of Neonates Born to Mothers With Severe Acute Respiratory Syndrome Coronavirus 2 Infection at a Large Medical Center in New York City. *JAMA Pediatr.* 2021;175(2):157-167.

22. Raschetti R, Vivanti AJ, Vauloup-Fellous C, Loi B, Benachi A, De Luca D. Synthesis and systematic review of reported neonatal SARS-CoV-2 infections. *Nat Commun.* 2020;11(1):5164.

For peer review only

23. De Bernardo G, Giordano M, Zollo G, et al. The clinical course of SARS-CoV-2 positive neonates. *J Perinatol.* 2020;40(10):1462-1469.

24. Vivanti AJ, Vauloup-Fellous C, Prevot S, et al. Transplacental transmission of SARS-CoV-2 infection. *Nat Commun.* 2020;11(1):3572.

25. Information for Pediatric Healthcare Providers. In. Vol December. CDC Centers for Disease Control and Prevention2020 December.

26. Viner RM, Mytton OT, Bonell C, et al. Susceptibility to SARS-CoV-2 Infection Among Children and Adolescents Compared With Adults: A Systematic Review and Meta-analysis. *JAMA Pediatr.* 2021;175(2):143-156.

27. Shekerdemian LS, Mahmood NR, Wolfe KK, et al. Characteristics and Outcomes of Children With Coronavirus Disease 2019 (COVID-19) Infection Admitted to US and Canadian Pediatric Intensive Care Units. *JAMA Pediatr.* 2020;174(9):868-873.

28. Dong Y, Mo X, Hu Y, et al. Epidemiological Characteristics of 2143 Pediatric Patients With 2019 Coronavirus Disease in China. *Pediatrics.* 2020.

29. Mithal LB, Machut KZ, Muller WJ, Kociolek LK. SARS-CoV-2 Infection in Infants Less than 90 Days Old. *J Pediatr.* 2020;224:150-152.

30. Yang S, Bhatnagar R, Byrne J, Jelks A. Universal testing for severe acute respiratory syndrome coronavirus 2 upon admission to three labor and delivery units in Santa Clara County, CA. *Clinical Journal of Obstetrics and Gynecology.* 2020;3:109-113.

31. Halscott TM, MS, Vaught JM, Miller EM, MPH. Management Considerations for Pregnant Patients with Covid-19. 2020;October 9. https://www.smfm.org/covidclinical.

32. Lynch KL, Whitman JD, Lacanienta NP, et al. Magnitude and Kinetics of Anti-Severe Acute Respiratory Syndrome Coronavirus 2 Antibody Responses and Their Relationship to Disease Severity. *Clin Infect Dis.* 2021;72(2):301-308.

33. Fouda GG, Martinez DR, Swamy GK, Permar SR. The Impact of IgG transplacental transfer on early life immunity. *Immunohorizons.* 2018;2(1):14-25.

34. Abu Raya B, Edwards KM, Scheifele DW, Halperin SA. Pertussis and influenza immunisation during pregnancy: a landscape review. *Lancet Infect Dis.* 2017;17(7):e209-e222.

35. Eberhardt CS, Blanchard-Rohner G, Lemaître B, et al. Maternal Immunization Earlier in Pregnancy Maximizes Antibody Transfer and Expected Infant Seropositivity Against Pertussis. *Clin Infect Dis.* 2016;62(7):829-836.

For peer review only

36. Wu YT, Liu J, Xu JJ, et al. Neonatal outcome in 29 pregnant women with COVID-19: A retrospective study in Wuhan, China. *PLoS Med.* 2020;17(7):e1003195.

37. Zeng H, Xu C, Fan J, et al. Antibodies in Infants Born to Mothers With COVID-19 Pneumonia. *JAMA.* 2020.

38. Dong L, Tian J, He S, et al. Possible Vertical Transmission of SARS-CoV-2 From an Infected Mother to Her Newborn. *JAMA.* 2020.

39. Shah PS, Diambomba Y, Acharya G, Morris SK, Bitnun A. Classification system and case definition for SARS-CoV-2 infection in pregnant women, fetuses, and neonates. *Acta Obstet Gynecol Scand.* 2020;99(5):565-568.

40. Lamouroux A, Attie-Bitach T, Martinovic J, Leruez-Ville M, Ville Y. Evidence for and against vertical transmission for severe acute respiratory syndrome coronavirus 2. *Am J Obstet Gynecol.* 2020;223(1):91.e91-91.e94.

41. Fenizia C, Biasin M, Cetin I, et al. Analysis of SARS-CoV-2 vertical transmission during pregnancy. *Nat Commun.* 2020;11(1):5128.

42. Kimberlin DW, Stagno S. Can SARS-CoV-2 Infection Be Acquired In Utero?: More Definitive Evidence Is Needed. *JAMA.* 2020.

For peer review only

Figure 1: Study participants enrollment

Figure 2: Temporal distribution of maternal and cord blood IgM and IgG

Panel A, B. scatterplots show the distribution of maternal blood SARS-CoV-2 IgM and IgG levels in relative fluorescent unit (RFU) at the time of delivery in Y-axis and days from maternal first positive SARS-CoV-2 PCR test to delivery in X-axis. Panel C shows the box plot of the distribution of the maternal IgG levels at the time of delivery in the maternal groups based on the number of days between maternal infection and delivery. The box represents the inter quartile range from 25th – 75th percentile (IQR). The marker within the box is the median and the "whiskers" reach the 1.5 times IQR. Panel D scatterplots show the distribution of cord blood SARS-CoV-2 IgG levels in RFU at the time of delivery in Y-axis and days from maternal first positive SARS-CoV-2 PCR test to delivery in X-axis. The different colors represent the severity of the maternal symptoms at the time of diagnosis.

Figure 3: Correlation of cord blood and maternal IgG and distribution of IgG transplacental transfer ratio

Panel A. Scatterplot shows the correlation of cord blood SARS-CoV-2 IgG levels in Y-axis and maternal blood SARS-CoV-2 IgG levels in X-axis in relative fluorescent unit (RFU). Panel B. Scatterplot shows the distribution of IgG transplacental ratio (cord blood/maternal blood SARS-CoV-2 IgG levels) in the Y-axis and days from maternal first positive SARS-CoV-2 PCR test to delivery in X-axis. Panel C. Scatterplot shows the distribution of IgG transplacental ratio in the Y-axis and gestational age at the time of delivery in X-axis. Panel D. Scatterplot shows the distribution of IgG transplacental ratio in the Y-axis and gestational age at the time of maternal first positive SARS-CoV-2 PCR test in X-axis. The different colors represent the severity of the maternal symptoms at the time of diagnosis.

Figure 4: Longitudinal follow-up of SARS-CoV-2 antibody levels in infants

Panel A shows the longitudinal IgG levels of the infants who had cord blood IgG level >50 relative fluorescent unit (RFU). The infants' IgG levels in RFU is shown in Y-axis, and the age of the infant in

For peer review only

weeks at the time of follow-up is shown in X-axis. The infants whose IgG became negative, <50RFU, during the longitudinal follow up are shown in red color. Panel B shows the IgG and IgM levels of the term infant whose cord antibody was negative and seroconverted at 2 weeks of life. Panel C shows the IgG and IgM levels of the 31 weeks preterm infant with confirmed intrapartum SARS-CoV-2 infection whose cord antibody was negative and seroconverted at 2 weeks of life.

For peer review only

Table 1. Maternal and neonatal demographics and outcomes

	**Maternal and infant serology cohort**
Mothers, n	145
Newborns, n	147
**Maternal demographics and outcomes**	
Maternal age, years, median (range)	27 (16, 42)
Gravida [a], median (range)	3 (1, 12)
Para [b], median (range)	1 (0, 9)
Hispanic, n (%)	126 (87)
Race	
White, n (%)	130 (90)
Black, n (%)	6 (4)
Asian, n (%)	9 (6)
Asymptomatic, n (%)	59 (41)
Mild to moderately symptomatic, n (%)	78 (54)
Severe to critically symptomatic, n (%)	8 (6)
Symptomatic at the time of delivery, n (%)	22 (15)
Cesarean section, n (%)	46 (32)
Multiple pregnancies, n (%)	3 (2)
Maternal diabetes, n (%)	29 (20)
Maternal hypertension, n (%)	30 (21)
Maternal obesity, n (%)	33 (23)
Preterm delivery, n (%)	15 (10)
Intrauterine fetal demise, n (%)	1 (1)

For peer review only

Neonatal demographics and outcomes	
Gestational age, weeks, median (range)	39.1 (27.4, 41.6)
Birth weight, grams, median (range)	3285 (990, 4670)
Breastfeeding in the hospital, n (%)	143 (97)
Exclusive breastfeeding in the hospital, n (%)	85 (58)
Rooming in with mother, n (%)	132 (90)
NICU admission, n (%) [c]	23 (16)
Length of stay during birth hospital, days, median (range)	2 (1, 81)
SARS-CoV-2 nasopharyngeal swab positive, n (%) [d]	1 (1)

[a]Gravida – number of pregnancies, [b]Para – number of deliveries
[c]Reasons for NICU admissions: 7 for prematurity, 1 for a congenital anomaly, 1 for dehydration and 14 for respiratory distress, metabolic acidosis and or evaluation for infection.
[d]SARS-COV-2 PCR using nasopharyngeal specimens was performed in 70 (99%) of the newborns born to mothers who were first PCR positive within two weeks of delivery.

For peer review only

Table 2. Maternal and cord blood serology and timing of maternal first positive PCR

	Total	0-13d	14-59d	60-179d	≥180d
**Maternal serology, n**	**129**	**56**	**28**	**36**	**9**
IgM- and IgG-, n	45	35	4	5	1
IgM+ and IgG-, n	4	4	0	0	0
IgM+ and IgG+, n	29	6	13	9[a]	1[b]
IgM- and IgG+, n	51	11	11	22	7
IgM+ and/or IgG+, n	84	21	24	31	8
IgM, RFU, median (range)	27 (7, 1388)	25·5 (2, 315)	34.5 (7, 1388)	26.5 (11, 263)	25 (7, 59)
IgG, RFU, median (range)	84 (1, 3582)	22·5 (1, 401)	178 (1, 1123)	194.5 (22, 2311)	199 (41, 3582)
**Cord blood serology, n**	**144**	**70**	**27**	**38**	**9**
IgG-, n	61	48	8	4	1
IgG+, n	83	22	18	32	8
IgG, RFU, median (range)	66.5 (0, 2916)	14 (0, 1820)	77 (2, 1164)	232 (22, 2916)	209 (45, 1173)
**Paired cord and maternal blood serology, n**	**125**	**54**	**26**	**36**	**9**
Maternal IgG + and Cord blood IgG +, n	69	12	19	31	7
Maternal IgG + and cord blood IgG -, n	8	4	3	0	1
Maternal IgG – and cord blood IgG -, n	45	37	4	4	0
Maternal IgG – and cord blood IgG, +, n	3	1	0	1	1
Maternal IgM + and Cord blood IgM +, n	3	0	1	2	0

For peer review only

Maternal IgM + and cord blood IgM -, n	29	10	11	7	1
Maternal IgM – and cord blood IgM -, n	93	44	14	27	8

RFU=Relative fluorescent unit.
[a] All 9 mothers' first positive SARS-VoC-2 PCR were between 63 and 103 days before delivery.
[b] This mother's SARS-VoC-2 PCR was positive at 10 weeks gestation and was positive again at the time of delivery at 39 weeks gestation.

For peer review only

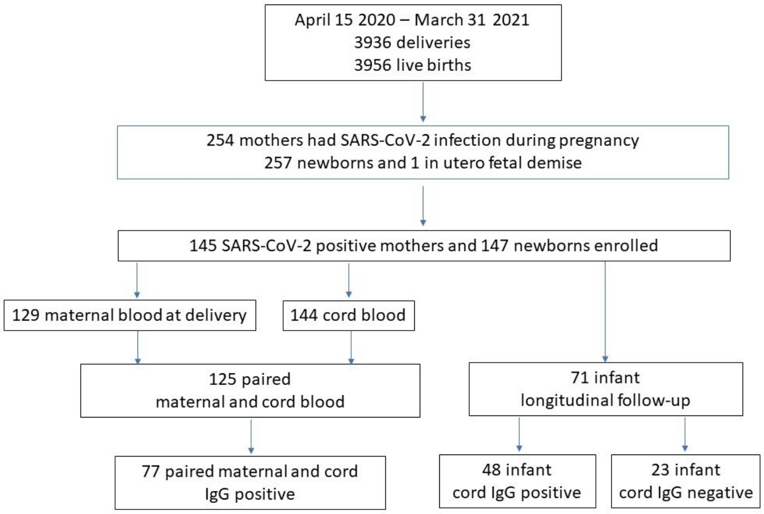

Figure 1. Study participants enrollment

108x60mm (300 x 300 DPI)

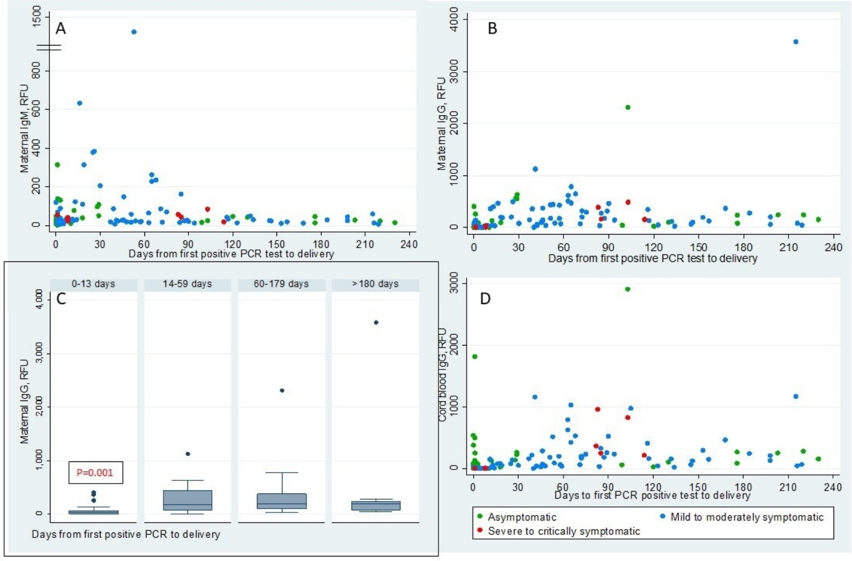

Figure 2. Temporal distribution of maternal and cord blood IgM and IgG
Panel A, B. scatterplots show the distribution of maternal blood SARS-CoV-2 IgM and IgG levels in relative fluorescent unit (RFU) at the time of delivery in Y-axis and days from maternal first positive SARS-CoV-2 PCR test to delivery in X-axis. Panel C shows the box plot of the distribution of the maternal IgG levels at the time of delivery in the maternal groups based on the number of days between maternal infection and delivery. The box represents the inter quartile range from 25th – 75th percentile (IQR). The marker within the box is the median and the "whiskers" reach the 1.5 times IQR. Panel D scatterplots show the distribution of cord blood SARS-CoV-2 IgG levels in RFU at the time of delivery in Y-axis and days from maternal first positive SARS-CoV-2 PCR test to delivery in X-axis. The different colors represent the severity of the maternal symptoms at the time of diagnosis.

108x60mm (300 x 300 DPI)

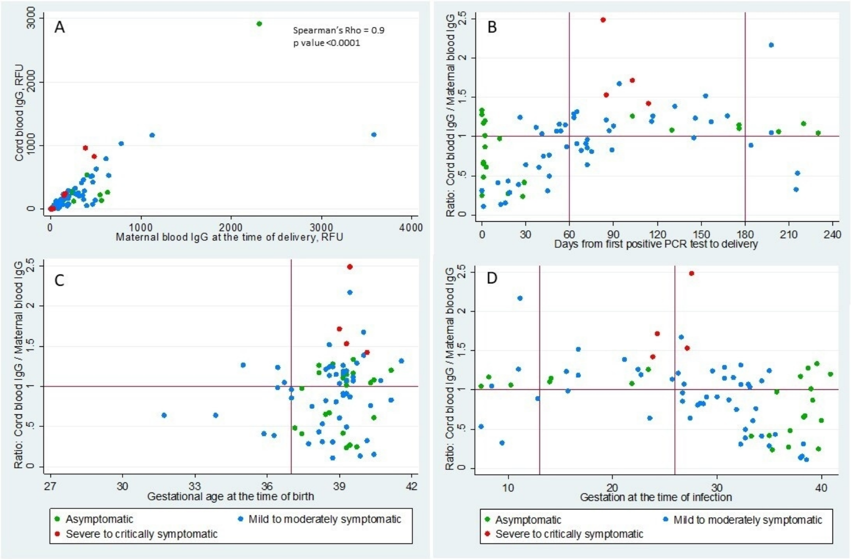

Figure 3. Correlation of cord blood and maternal IgG and distribution of IgG transplacental transfer ratio Panel A. Scatterplot shows the correlation of cord blood SARS-CoV-2 IgG levels in Y-axis and maternal blood SARS-CoV-2 IgG levels in X-axis in relative fluorescent unit (RFU). Panel B. Scatterplot shows the distribution of IgG transplacental ratio (cord blood/maternal blood SARS-CoV-2 IgG levels) in the Y-axis and days from maternal first positive SARS-CoV-2 PCR test to delivery in X-axis. Panel C. Scatterplot shows the distribution of IgG transplacental ratio in the Y-axis and gestational age at the time of delivery in X-axis. Panel D. Scatterplot shows the distribution of IgG transplacental ratio in the Y-axis and gestational age at the time of maternal first positive SARS-CoV-2 PCR test in X-axis. The different colors represent the severity of the maternal symptoms at the time of diagnosis.

108x60mm (300 x 300 DPI)

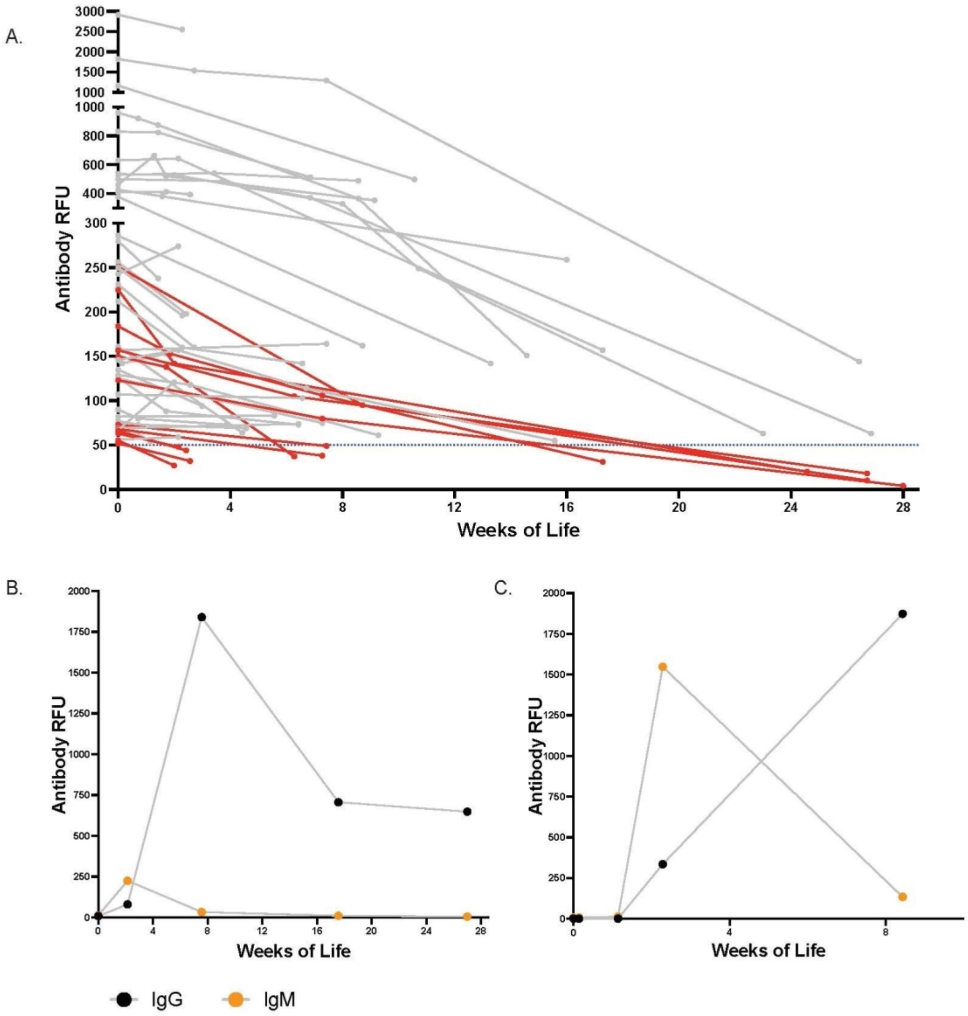

Figure 4. Longitudinal follow-up of SARS-CoV-2 antibody levels in infants
Panel A shows the longitudinal IgG levels of the infants who had cord blood IgG level >50 relative fluorescent unit (RFU). The infants' IgG levels in RFU is shown in Y-axis, and the age of the infant in weeks at the time of follow-up is shown in X-axis. The infants whose IgG became negative, <50RFU, during the longitudinal follow up are shown in red color. Panel B shows the IgG and IgM levels of the term infant whose cord antibody was negative and seroconverted at 2 weeks of life. Panel C shows the IgG and IgM levels of the 31 weeks preterm infant with confirmed intrapartum SARS-CoV-2 infection whose cord antibody was negative and seroconverted at 2 weeks of life.

140x143mm (300 x 300 DPI)

PCR Methods:

Nasopharyngeal swab PCR assays.
The PCR test was performed using the following four assays that have been validated and used for clinical diagnostic purpose in our hospital: Xpert® Xpress SARS-CoV-2 assay (Cepheid, Sunnyvale, California, USA), DiaSorin Simplexa™ COVID-19 Direct assay (Diasorin Molecular, Cypress, California, USA), Perkin Elmer® nCoV NAD assay (Perkin Elmer, Waltham, Massachusetts, USA), and Hologic® Aptima™ SARS-CoV-2 Assay (Hologic Inc., Marlborough, Massachusetts, USA).

RNA Extraction**.** Maternal blood, cord blood, placental tissue, and infant meconium RNA was extracted using the QIAmp Viral RNA Mini Kit following the manufacturer's instructions with some adjustments. 300uL of maternal and cord blood in RNAlater (1:1.3 ratio) were used for each extraction. 15-25 mg of placenta and 300 μg of meconium in viral transport media was used for extraction. The kit protocol was followed with buffer amounts scaled up proportionally for the starting amount. RNA was eluted in a 40uL elution buffer for blood and 20uL elution buffer for placenta and meconium. RNA quantity was measured using the Qubit RNA High Sensitivity Assay Kit.

Quantitative real-time PCR**.** Quantitative polymerase chain reaction was performed using the ABI StepOne Plus system. Primer sequences targeted the N (nucleotide) and Orf1b (ORF1b-nsp14) gene. Primer sequences are as follows: forward primer targeting N gene \ (HKU-NF): 5'-TAATCAGACAAGGAACTGATTA-3';Reverse primer (HKU-NR): 5'-CGAAGGTGTGACTTCCATG-3'; and Probe (HKU-NP): 5'-FAM-GCAAATTGTGCAATTTGCGG-TAMRA-3'. Forward primer targeting Orf1b-nsp14 gene (HKU-ORF1b-nsp14F): 5'-TGGGGYTTTACRGGTAACCT-3'; Reverse primer (HKU- ORF1b-nsp14R): 5'-AACRCGCTTAACAAAGCACTC-3'; and Probe (HKU-ORF1b-nsp141P): 5'-FAM-TAGTTGTGATGCWATCATGACTAG-TAMRA-3. RT-qPCR reactions were performed using the TaqMan Fast Virus 1-step Master Mix according to the manufacturer's instructions.

Table 1: PCR Reagents

Reagent	Volume per rxn (μL)
Water (RNAse free)	7.5
TaqMan Fast Virus 1-step (4X)	5
Forward Primer (10 μM)	1
Reverse Primer (10 μM)	1
Prove (10 μM)	0.5
RNA Sample	5

Table 2: PCR Cycle

Steps	Temperature (C)	Time (mm:ss) # cycles
Reverse Transcription	50	05:00
RT Inactivation/denaturation	96	00:20
Amplification	95	00:05:40
Amplification	60	00:30

Table 3: Distribution of severity of maternal symptoms at the time of diagnosis

	**Asymptomatic mothers**	**Mild-moderately symptomatic mothers**	**Severe-critically symptomatic mothers**
	**59**	**78**	**8**
**Time between maternal infection and delivery**			
<60 days, n	50	46	3
60-180 days, n	6	26	5

For peer review only - http://bmjopen.bmj.com/site/about/guidelines.xhtml

>180 days, n	3	6	0
**Trimester at the time of maternal infection**			
First Trimester, n	3	8	0
Second Trimester, n	7	12	0
Third Trimester, n	49	58	8
**Trimester at the time of delivery**			
First Trimester, n	0	0	0
Second Trimester, n	0	0	0
Third Trimester, n	59	78	8

Table 4: Delivery specimen PCR results

Participant	Maternal Blood	Cord Blood	Placenta	Infant Meconium
#1, cord IgM 62 RFU	Negative	Negative	Negative	-
#2, cord IgM 65 RFU	-	Negative	Negative	-
#3, cord IgM 136 RFU	-	-	Negative	Negative

For peer review only

STROBE 2007 (v4) checklist of items to be included in reports of observational studies in epidemiology*
Checklist for cohort, case-control, and cross-sectional studies (combined)

Section/Topic	Item #	Recommendation	Reported on page #
**Title and abstract**	1	(*a*) Indicate the study's design with a commonly used term in the title or the abstract	3
		(*b*) Provide in the abstract an informative and balanced summary of what was done and what was found	3
**Introduction**			
Background/rationale	2	Explain the scientific background and rationale for the investigation being reported	7-9
Objectives	3	State specific objectives, including any pre-specified hypotheses	9
**Methods**			
Study design	4	Present key elements of study design early in the paper	9
Setting	5	Describe the setting, locations, and relevant dates, including periods of recruitment, exposure, follow-up, and data collection	9-11
Participants	6	(*a*) *Cohort study*—Give the eligibility criteria, and the sources and methods of selection of participants. Describe methods of follow-up<br>*Case-control study*—Give the eligibility criteria, and the sources and methods of case ascertainment and control selection. Give the rationale for the choice of cases and controls<br>*Cross-sectional study*—Give the eligibility criteria, and the sources and methods of selection of participants	9,10 Figure 1
		(*b*) *Cohort study*—For matched studies, give matching criteria and number of exposed and unexposed<br>*Case-control study*—For matched studies, give matching criteria and the number of controls per case	
Variables	7	Clearly define all outcomes, exposures, predictors, potential confounders, and effect modifiers. Give diagnostic criteria, if applicable	9,10
Data sources/ measurement	8*	For each variable of interest, give sources of data and details of methods of assessment (measurement). Describe comparability of assessment methods if there is more than one group	9-11, supplemental Methods, supplemental Tables 1 and 2
Bias	9	Describe any efforts to address potential sources of bias	10
Study size	10	Explain how the study size was arrived at	Figure 1
Quantitative variables	11	Explain how quantitative variables were handled in the analyses. If applicable, describe which groupings were chosen and why	11
Statistical methods	12	(*a*) Describe all statistical methods, including those used to control for confounding	11
		(*b*) Describe any methods used to examine subgroups and interactions	11

For peer review only

		(*c*) Explain how missing data were addressed	10
		(*d*) *Cohort study*—If applicable, explain how loss to follow-up was addressed<br>*Case-control study*—If applicable, explain how matching of cases and controls was addressed<br>*Cross-sectional study*—If applicable, describe analytical methods taking account of sampling strategy	10
		(*e*) Describe any sensitivity analyses	
**Results**			
Participants	13*	(a) Report numbers of individuals at each stage of study—eg numbers potentially eligible, examined for eligibility, confirmed eligible, included in the study, completing follow-up, and analysed	Figure 1, 12-15,
		(b) Give reasons for non-participation at each stage	Figure 1
		(c) Consider use of a flow diagram	Figure 1
Descriptive data	14*	(a) Give characteristics of study participants (eg demographic, clinical, social) and information on exposures and potential confounders	11, Table 1
		(b) Indicate number of participants with missing data for each variable of interest	Figure 1, Table 2
		(c) *Cohort study*—Summarise follow-up time (eg, average and total amount)	Figure1,Table2, 14
Outcome data	15*	*Cohort study*—Report numbers of outcome events or summary measures over time	Figure 1-4 Table,12-14,
		Case-control study—Report numbers in each exposure category, or summary measures of exposure	
		Cross-sectional study—Report numbers of outcome events or summary measures	
Main results	16	(*a*) Give unadjusted estimates and, if applicable, confounder-adjusted estimates and their precision (eg, 95% confidence interval). Make clear which confounders were adjusted for and why they were included	12,13
		(*b*) Report category boundaries when continuous variables were categorized	Table 2, 12,13
		(*c*) If relevant, consider translating estimates of relative risk into absolute risk for a meaningful time period	
Other analyses	17	Report other analyses done—eg analyses of subgroups and interactions, and sensitivity analyses	Table 2, 12-14
**Discussion**			
Key results	18	Summarise key results with reference to study objectives	14
Limitations	19	Discuss limitations of the study, taking into account sources of potential bias or imprecision. Discuss both direction and magnitude of any potential bias	18
Interpretation	20	Give a cautious overall interpretation of results considering objectives, limitations, multiplicity of analyses, results from similar studies, and other relevant evidence	15-19
Generalisability	21	Discuss the generalisability (external validity) of the study results	18
**Other information**			
Funding	22	Give the source of funding and the role of the funders for the present study and, if applicable, for the original study on which the present article is based	4

For peer review only

*Give information separately for cases and controls in case-control studies and, if applicable, for exposed and unexposed groups in cohort and cross-sectional studies.

Note: An Explanation and Elaboration article discusses each checklist item and gives methodological background and published examples of transparent reporting. The STROBE checklist is best used in conjunction with this article (freely available on the Web sites of PLoS Medicine at http://www.plosmedicine.org/, Annals of Internal Medicine at http://www.annals.org/, and Epidemiology at http://www.epidem.com/). Information on the STROBE Initiative is available at www.strobe-statement.org.

For peer review only
